# Sequence-selective dynamic covalent assembly of information-bearing oligomers

**DOI:** 10.1038/s41467-020-14607-3

**Published:** 2020-02-07

**Authors:** Samuel C. Leguizamon, Timothy F. Scott

**Affiliations:** 1 Department of Chemical Engineering, University of Michigan, Ann Arbor, MI 48109 USA; 2 Macromolecular Science and Engineering Program, University of Michigan, Ann Arbor, MI 48109 USA; 3 Department of Chemical Engineering, Monash University, Clayton, VIC 3800 Australia; 4 Department of Materials Science and Engineering, Monash University, Clayton, VIC 3800 Australia

**Keywords:** Polymer synthesis, Polymers

## Abstract

Relatively robust dynamic covalent interactions have been employed extensively to mediate molecular self-assembly reactions; however, these assembly processes often do not converge to a thermodynamic equilibrium, instead yielding mixtures of kinetically-trapped species. Here, we report a dynamic covalent self-assembly process that mitigates kinetic trapping such that multiple unique oligomers bearing covalently coreactive pendant groups are able to undergo simultaneous, sequence-selective hybridization with their complementary strands to afford biomimetic, in-registry molecular ladders with covalent rungs. Analogous to the thermal cycling commonly employed for nucleic acid melting and annealing, this is achieved by raising and lowering the concentration of a multi-role reagent to effect quantitative dissociation and subsequently catalyze covalent bond rearrangement, affording selective assembly of the oligomeric sequences. The hybridization specificity afforded by this process further enabled information encoded in oligomers to be retrieved through selective hybridization with complementary, mass-labeled sequences.

## Abstract (web summary)

Dynamic covalent interactions have been employed to mediate molecular self-assembly reactions but often do not converge to a thermodynamic equilibrium and yield a mixture of kinetically trapped species. Here, the authors show a sequence-selective, dynamic covalent self-assembly process that mitigates kinetic trapping to afford biomimetic molecular ladders with covalent rungs.

## Introduction

The capacity for sequence-specific polymer strands to selectively assemble into intricate, folded structures and multimeric complexes relies upon the information borne by their residue sequences^1,2^. Although there are several examples of the non-covalent assembly of information-bearing species^3–6^, nucleic acids represent a particularly versatile class for producing nanostructures which, through careful consideration of their nucleobase sequence, can be designed to predictably self-assemble via the hybridization of complementary strands into arbitrary structures with nanometer-scale precision^7–10^. Unfortunately, the versatility of nucleic acids as nanoconstruction media is tempered by the thermal and mechanical instability of the resultant structures, attributable to the weakness of the hydrogen bonds that hold the strands together^11^. Efforts to improve the stability of double-stranded, nucleic acid-like duplexes include the utilization of abiotic, neutral backbones^12–14^, the integration of isostere nucleobase mimetics^15,16^, and the incorporation of photo-induced cross-links^17,18^; nevertheless, the assembly processes for these duplexes remain mediated by intermolecular interactions. In contrast, employing dynamic covalent interactions to mediate molecular self-assembly processes can afford inherently robust, covalently cross-linked structures^19–26^, although impediments to connectivity rearrangement amongst the assembled components, including slow reaction rates and reaction site inaccessibility, often result in the kinetic trapping of non-equilibrium species^27–29^.

Here, we describe a dynamic covalent assembly approach where mixtures of sequence-specific oligomers bearing covalently coreactive amine-based and aldehyde-based pendant groups selectively associate and dimerize with their oligomeric complements to afford in-registry molecular ladders with imine-based covalent rungs, mimicking the selective, information-directed hybridization of complementary nucleic acid sequences.

## Results

### Dynamic covalent assembly of encoded molecular ladders

Peptoids (i.e., oligo(*N*-substituted glycine)s)^30^ were employed here as the precursor oligomers owing to their ready synthetic accessibility via Zuckerman’s sub-monomer solid phase synthetic method^31^. Moreover, the peptoid backbone is inherently neutral, achiral, and lacks hydrogen bond donor sites^32^, ensuring that oligomer hybridization selectivity is dominated by covalent interactions between pendant groups. These oligomers were synthesized as binary sequences of amine and aldehyde pendant groups (denoted here as ‘1’ and ‘0’, respectively) alternating with inert spacer residues such that, owing to the zig-zag ‘Σ-strand’ conformation adopted by the constituent peptoid chains, the reactive groups were presented on the same side of the peptoid backbone^33^. The spacer residues also increased solubility of both the precursor oligomers and hybridized molecular ladders, preventing precipitation during the self-assembly process. To enable facile identification of the hybridization product species by mass spectrometry, sequences were mass-tagged by adding extra, inert spacer residues to the peptoid ends during synthesis. Additionally, each imine bond generated by an amine/aldehyde condensation reaction reduces the molar mass of the resultant molecular ladder by 18 g/mol owing to the loss of a water molecule, enabling in- and out-of-registry species to be readily distinguished by mass. As the oligomer sequences bore multiple, covalently coreactive functional group types, ethylene acetals were used as aldehyde-protecting groups to preclude premature reaction between coreactive pendant groups during synthesis and purification, and which could then be deprotected in situ, allowing the dynamic covalent assembly reaction to proceed. Monomer residue structures are as shown in Supplementary Table 1 and purified, acetal-protected peptoid sequences were characterized as shown in Supplementary Figs. 1–3.

**Fig. 1:**
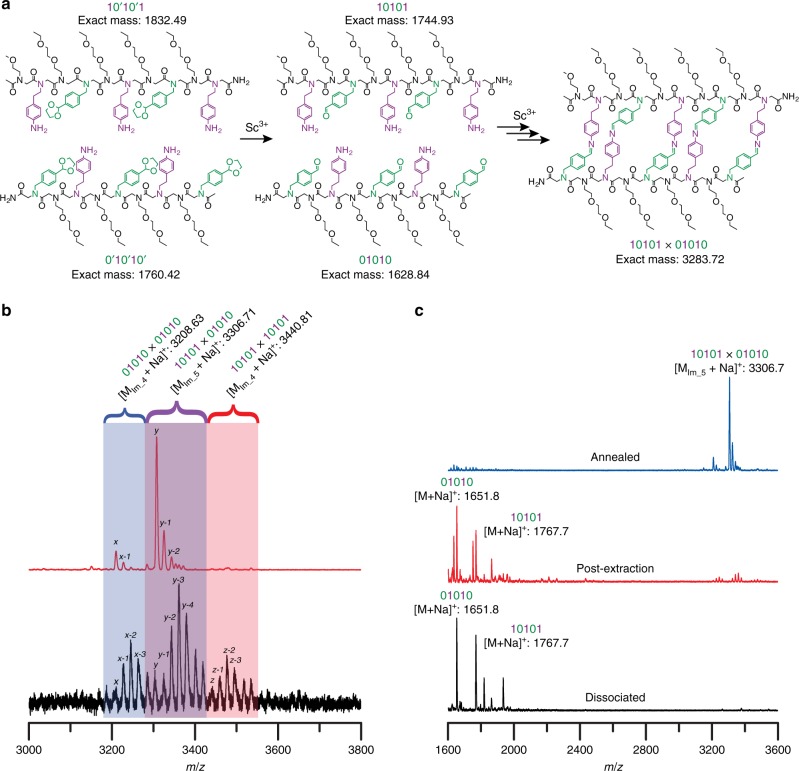
Dynamic covalent assembly of encoded molecular ladders. **a** Structures of complementary, mass-labeled precursor peptoids and desired, in-registry molecular ladder product. **b** MALDI mass spectra of molecular ladder reaction mixtures generated either after a single-step, deprotection, and direct assembly process (bottom, black), or via a stepwise, dissociation/extraction/annealing assembly process (top, red). Peaks at multiples of +18*m*/*z* values are attributable to ladders species with fewer rungs (e.g., *y* = in-registry, 5-rung 10101 × 01010 molecular ladder, *y*−1 = out-of-registry, 4-rung 10101 × 01010 molecular ladder, etc.). **c** MALDI mass spectra of the reaction mixture initially containing 10101 and 01010 sequences and a high loading of Sc(OTf)_3_ (bottom, black), immediately after aqueous extraction (middle, red), and after annealing at 70 °C for 2 h (top, blue). Expected exact masses: [M_01010_ + Na]^+^ = 1651.83; [M_10101_ + Na]^+^ = 1767.92; [M_01010×__01010_ + Na]^+^ (*x*) = 3208.63; [M_10101×__01010_ + Na]^+^ (*y*) = 3306.71; [M_10101×__10101_ + Na]^+^ (*z*) = 3440.81.

Sequence-selective, dynamic covalent oligomer hybridization was initially examined using a model system consisting of two complementary peptoid sequences, each bearing alternating amine-based and aldehyde-based reactive pendant groups (i.e., sequences 10101, mass-labeled with an additional methoxyethylamine residue at its *N*-terminal end, and 01010, see Fig. 1a). Our group has previously demonstrated the utilization of scandium(III) triflate at low loadings as a dual-role, Lewis acidic catalyst both to effect the in situ deprotection of oligomer-borne, acetal-protected aldehydes and to catalyze transimination and imine metathesis reactions for the rearrangement of imine bonds generated by amine/aldehyde condensation reactions, thereby successfully yielding molecular ladders with up to eight rungs via the dynamic covalent assembly of diblock, amine-bearing and aldehyde-bearing precursor oligomers. Consequently, we applied the previously determined reaction conditions (i.e., 0.2 equivalents of Sc(OTf)_3_ for 2 h at 70 °C)^34^ to the model system examined here.

For this system, the anticipated, thermodynamically favored product should emerge from the cross-reaction between the 10101 sequence and its 01010 oligomeric complement (i.e., 10101 × 01010) to selectively afford the formation of an in-registry molecular ladder with five imine-based rungs (denoted here as Im_5). As determined by MALDI mass spectrometry (Fig. 1b, bottom spectrum), the applied reaction conditions did yield complete acetal deprotection; however, these conditions resulted in the non-selective, kinetically trapped generation of multiple in- and out-of-registry molecular ladder species resulting from the incomplete coreaction of two 01010 (01010 × 01010) and two 10101 (10101 × 10101) sequences, both of which can yield molecular ladders with a maximum of four imine rungs (i.e., Im_4), in addition to those from the 10101 × 01010 cross-reaction.

Our previous work on the Sc(III)-catalyzed dynamic covalent assembly of imine-based molecular ladders revealed that the assembly reaction proceeds via a hybrid mechanism, where amine-bearing and aldehyde-bearing oligomers initially associate and bind through condensation reactions at any point along the backbone to form out of registry molecular ladders, whereupon transimination and imine metathesis reactions proceed to shuffle the imine bonds until the molecular ladders come into registry^35^. Whereas this mechanism enabled the annealing of out-of-registry, intermediate species for simple systems composed of homo- or diblock oligomers, more complex sequences where the reactive residue type switches between amine and aldehyde pendant groups multiple times impede inter-strand bond shuffling as imine rearrangement reactions can no longer occur exclusively between adjacent residues. Rather, rearrangement reactions for such sequences would need to proceed between relatively remote functionalities to enable inter-strand shuffling. This registry mechanism is dissimilar to that commonly employed for DNA assembly where, instead of inter-strand shuffling, dissociated DNA sequences selectively hybridize with their complements upon gradual cooling from raised temperature to afford in-registry double helices at low error rates^8^. Pioneering work by the Lehn group on the catalysis of imine rearrangement reactions demonstrated that, at moderate concentrations, Lewis acidic rare-earth metal triflates do act to catalyze imine connectivity rearrangement, but at raised concentrations they shift the amine/aldehyde condensation reaction equilibrium towards the unpaired reactants^36^. Analogous to the thermal cycling commonly employed for nucleic acid melting and annealing, raising and lowering the scandium triflate concentration was thus used to mitigate kinetic trapping by enabling quantitative dissociation and subsequent, sequence-selective assembly of the 10101 and 01010 peptoid sequences to afford the desired 10101 × 01010 molecular ladder product (see Fig. 1b, top spectrum).

This process is detailed in Fig. 1c where MALDI mass spectra collected on reaction mixture aliquots after each process step are presented. The original reaction mixture initially contained a stoichiometric amount of ethylene acetal-protected 10101 and 01010 sequences and a high loading (i.e., 1.5 equivalents per amine/aldehyde pair) of Sc(OTf)_3_. Under these reaction conditions, the acetals groups were quantitatively deprotected to reveal amine-reactive aldehydes; nevertheless, the peptoid strands remained dissociated, evident from the presence of deprotected single strands at 1651.8*m*/*z* and 1767.7*m*/*z* and virtual absence of paired species from 3200–3600*m*/*z*, owing to the influence of Sc(OTf)_3_ on the reaction equilibrium (Fig. 1c, bottom black spectrum). This reaction mixture was subject to an aqueous extraction to selectively extract a fraction of the Sc(OTf)_3_, reducing its relative loading. The residual scandium concentration was determined by inductively coupled plasma mass spectrometry (ICP-MS), revealing that 0.055 ± 0.002 equivalents of Sc^3+^ remained post-extraction, approaching the optimal concentration for transimination catalysis reported previously^36^. Characterization of the reaction mixture by mass spectrometry immediately after extraction indicated that the unpaired single strands remained dominant, although non-negligible amounts of intermediate hybridized species were observable (Fig. 1c, middle red spectrum). Owing to the chemical and molecular weight similarities between in- and out-of-registry ladders, ionization efficiencies in MALDI mass spectrometry are presumed sufficiently similar to permit relative concentrations of the individual species to be estimated^35,37,38^. Heating for 2 h at 70 °C to anneal the system yielded the in-registry 10101 × 01010 molecular ladder as the primary reaction product (Fig. 1c, top blue spectrum). Small peaks attributable to 01010 × 01010 ladder species remained after annealing owing to a slight stoichiometric imbalance that arose during the extraction step where some of the 10101 sequence was lost in the aqueous extraction. Additionally, trace amounts of species at +18*m*/*z* (i.e., 4-rung ladders) were attributable to the residual Sc^3+^ affecting the reaction equilibrium rather than kinetically trapped, out-of-registry products. Notably, when the system was annealed at room temperature, fully in-registry 10101 × 01010 ladder species were observed by MALDI mass spectrometry within 1 day and emerged as the major peak within 7 days (Supplementary Fig. 4) indicating that, although raised temperatures increase reaction rates as expected, the self-assembly process does proceed under ambient conditions. Gel permeation chromatography (GPC), calibrated using low dispersity polystyrene standards, confirmed formation of the 10101 × 01010 molecular ladder as the predominant product (43.8%) by comparing elution volumes of inert, Alloc-protected single strands to hybridization mixtures (Supplementary Fig. 5a–d). Notably, this yield is lower than previously determined yields for imine rung-based molecular ladders assembled from homoblock oligomeric sequences^39^ as these oligomers are incapable of participating in intramolecular amine/aldehyde condensation reactions, thus eliminating a potential mechanism for side product generation. Peak fractions, collected during GPC purification, were characterized by MALDI mass spectrometry to identify the fraction constituents (Supplementary Fig. 5e). The hybridization product was preceded by several minor peaks at higher molecular weights, attributable to multimeric complexes, and proceeded by single-stranded species (Supplementary Fig. 5f and g), including unreacted 10101 and 01010 strands and those with either one intramolecular imine bond to yield macrocycles, or two intramolecular imine bonds to potentially afford hairpin-like structures. The occurrence of a binding event was further verified with diffusion ordered spectroscopy (DOSY) NMR, which demonstrated a decrease in the diffusion coefficient after extraction and annealing of an initially dissociated mixture containing 10101 and 01010 strands (Supplementary Fig. 6).

To examine the effect of Sc^3+^ concentration on molecular ladder dissociation, varying amounts of scandium triflate were added to post-annealed, in-registry 10101 × 01010 reaction mixtures which were then heated at 60 °C for 6 h, left to stand at room temperature overnight to ensure system equilibration, and characterized by mass spectrometry (Supplementary Fig. 7). At 0.20 equivalents of Sc(OTf)_3_ (matching the Sc^3+^ concentration employed for the single-pot, single-step assembly method), the desired in-registry molecular ladder remained the major product; however, peaks at multiples of +18*m*/*z*, attributable to ladders species with fewer rungs, were more apparent, suggesting a significant shift in the reaction equilibrium towards the amine and aldehyde reactants. At 0.30 equivalents of Sc^3+^ equivalents and above, the extent of ladder dissociation progressively increases and peaks attributable to dimerized species of mismatched, non-complementary strands (i.e., those consisting of 01010 × 01010 and 10101 × 10101 sequences) become more prevalent.

To examine the robustness of this dissociation/extraction/annealing assembly approach to alternative, Lewis acidic multi-role reagents, reaction mixtures of 10101 and 01010 oligomeric sequences were formulated with one of a library of rare-earth metal triflates with varying Lewis acidity, including Yb(OTf)_3_, Lu(OTf)_3_, Y(OTf)_3_, and Sm(OTf)_3_ (Supplementary Fig. 8). Whereas ytterbium triflate is more Lewis acidic than lutetium triflate, the annealing period required for in-registry 10101 × 01010 molecular ladder equilibrium was at least 3 days at 70 °C for the ytterbium triflate-containing reaction mixture while overnight at 70 °C was sufficient to yield in-registry ladders for the mixture formulated with lutetium triflate. The residual rare-earth metal triflate in the reaction mixtures post-extraction was assessed by ICP-MS and revealed that a greater concentration of lutetium triflate remained in the organic layer than that of ytterbium triflate (0.014 ± 0.0008 eq. and 0.009 ± 0.0004 eq., respectively, indicating that the higher residual concentration of Lu^3+^ more than compensated for its lower Lewis acidity than Yb^3+^. Notably, again owing to their lower Lewis acidity, three equivalents of yttrium triflate and samarium triflate (i.e., twice the required equivalents of Sc^3+^, Yb^3+^, and Lu^3+^) were necessary to fully deprotect and dissociate the oligomeric strands. After extraction, in-registry molecular ladder equilibrium was achieved after 7 days at 70 °C for yttrium triflate-containing solution, while the reaction mixture incorporating samarium triflate required an additional 2 days for complete ladder assembly. Here, ICP-MS revealed residual, post-extraction concentrations of 0.025 ± 0.001 eq. and 0.059 ± 0.003 eq. for Y^3+^ and Sm^3+^, respectively).

### Competitive dynamic covalent assembly

**Fig. 2:**
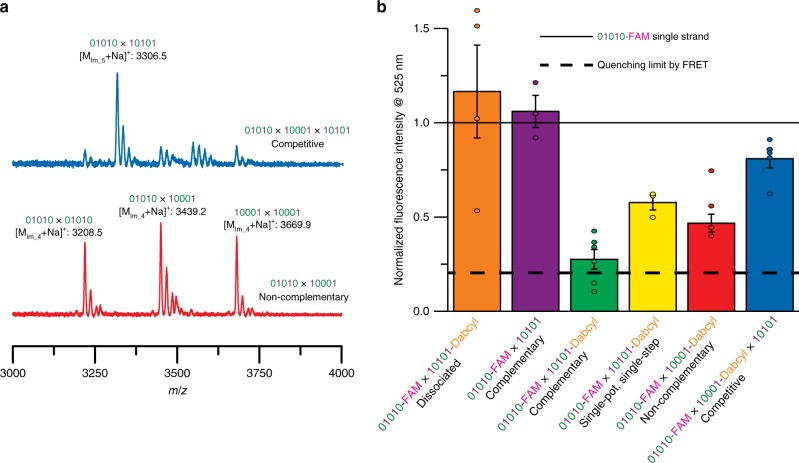
Competitive dynamic covalent assembly. **a** MALDI mass spectra of reaction mixtures generated after applying the dissociation/extraction/annealing process to non-complementary 01010 and 10001 sequences (bottom, red) and to a ternary mixture of a competitive environment consisting of a complementary sequence and a non-complementary sequence via the dissociation/extraction/annealing process (top, blue). Peaks at multiples of +18*m*/*z* values are attributable to ladders species with fewer rungs. **b** Sequence-selective hybridization assessed by FRET and normalized to the fluorescence intensity of a 01010-FAM sequence single strand. Assembly solutions were excited at 495 nm and fluorescence emission was measured at 425 nm. Error bars represent standard error. The horizontal solid and dashed lines 1 and 0.2, respectively, represent the unquenched 01010-FAM control and the reported FAM/DABCYL fluorescence quenching efficiency of 80%^40^, respectively.

The single reactant pair mismatch discrimination of our dynamic covalent self-assembly process was examined using combinations of peptoid strands, including the complementary pair of 10101 and 01010, and a 10001 sequence that is non-complementary with the other two. Importantly, the 10001 and 01010 sequences contain only a single mismatch located at the third residue to preclude any overhangs that might otherwise occur from influencing the 10001 × 01010 hybrid stability. Subjecting a non-complementary mixture of 10001 and 01010 strands in a 1:1 stoichiometric ratio to the dissociation/extraction/annealing reaction conditions yielded three cohorts of dimerized ladder species, 10001 × 10001, 01010 × 01010, and 01010 × 10001 (Fig. 2a, bottom red spectrum), demonstrating poor hybridization selectivity. In contrast, upon inclusion of a stoichiometric amount of the 10101 sequence to the initial reaction mixture to afford a ternary sequence system, where two of the peptoid strands are complementary (i.e., 10101 and 01010) results in the preferential generation of a complementary and in-registry 10101 × 01010 molecular ladder as the major mass spectrum peak (Fig. 2a, top blue spectrum).

Additional quantification of the hybridization selectivity was performed using peptoid strands functionalized with 5,6-carboxyfluorescein (FAM) and 4-((4-(dimethylamino)phenyl)azo)benzoyl (DABCYL) groups as a Förster resonance energy transfer (FRET) fluorophore and fluorescence quencher pair, respectively. These groups were selected for FRET studies owing to their ready incorporation as oligomer end groups and their stability to resin cleavage conditions. Here, the oligomer sequence 01010 was labeled with FAM (denoted as 01010-FAM) and the sequences 10001 and 10101 were labeled with DABCYL (10001-DABCYL and 10101-DABCYL, respectively). All assays were normalized to a non-quenched fluorescence intensity ceiling, determined using a solution of 01010-FAM as a single-strand negative control. A control solution incorporating 01010-FAM and 10101-DABCYL, fully dissociated with 1.5 eq. of scandium triflate, was assessed to confirm the absence of fluorescence quenching for un-hybridized strands, while another solution of 01010-FAM × 10101 (i.e., omitting a quencher) was examined to confirm that the hybridization of the FAM-bearing strand with its DABCYL-free complementary sequence does not negatively affect fluorescence (see Fig. 2b, orange and purple bars, respectively). Upon treatment with the dissociation/extraction/annealing process, the 01010-FAM × 10101-DABCYL reaction mixture afforded a normalized fluorescence intensity of 0.275 ± 0.050 (i.e., a 72.5 ± 5.0% reduction in fluorescence, see Fig. 2b, green bar), a value approaching the reported FAM/DABCYL fluorescence quenching efficiency of 80%^40^. Conversely, solutions of both the same 01010-FAM and 10101-DABCYL oligomer pair after treatment with the single-pot, single-step assembly method, and the non-complementary 01010-FAM and 10001-DABCYL sequences after the dissociation/extraction/annealing process yielded moderate reductions in fluorescence intensity (Fig. 2b, yellow and red bars, respectively); however, the extent of fluorescence quenching for these systems was curtailed by the generation not only of hybridized species bearing both FAM and DABCYL but also of hybrids exclusively bearing either FAM or DABCYL groups (i.e., equivalent to the hybridized species between non-complementary strands apparent in Fig. 1b, bottom black, and Fig. 2a, bottom red spectra, respectively). Finally, hybridization selectivity was examined using a ternary system, equivalent to that described above, employing stoichiometric amounts of the sequences 01010-FAM, 10001-DABCYL, and quencher-free 10101. After the dissociation/extraction/annealing process, the normalized fluorescence intensity was determined to be 0.810 ± 0.055 (Fig. 2b, blue bar), demonstrating the preferential hybridization of complementary sequences enabled by this process to selectively afford the 10101 × 01010-FAM molecular ladder bearing 5 imine-based rungs (Fig. 2b, green bar) over a non-complementary, 4 imine-rung 10001-DABCYL × 01010-FAM molecular ladder (Fig. 2b, red bar).

### Sequence-selective hybridization

**Fig. 3:**
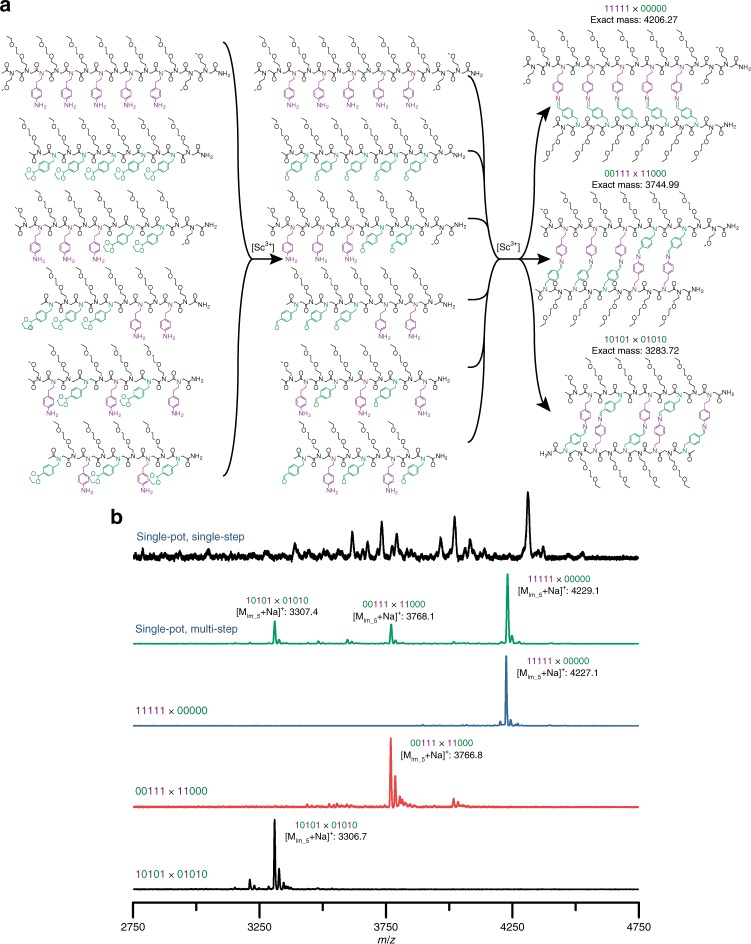
Sequence-selective hybridization. **a** Structures of mass-labeled, ethylene acetal-protected precursor peptoid sequences, deprotected, dissociated sequences upon treatment with Sc(OTf)_3_, and in-registry molecular ladders upon [Sc^3+^] extraction and annealing. **b** MALDI mass spectra of individual encoded molecular ladders assembled via the dissociation/extraction/annealing process, including 10101 × 01010 (bottom, black), 00111 × 11000 (second from bottom, red), 11111 × 00000 (middle, blue), and a single-pot solution of all six oligomers to yield three in-registry molecular ladders (second from top, green). A single-pot solution of the six oligomers after the single-step, deprotection and direct assembly process (top, black) is shown for comparison. Expected exact masses: [M_10101×__01010_ + Na]^+^ = 3306.71; [M_00111×__11000_ + Na]^+^ = 3767.93; [M_11111×__00000_ + Na]^+^ = 4229.25. Peaks at multiples of +18*m*/*z* values are attributable to ladders species with fewer rungs.

The assembly of sophisticated, multimeric macromolecular complexes requires the selective and concurrent hybridization of multiple information-encoded components^7–10^. To examine the potential for this dynamic covalent assembly system to achieve concurrent hybridization selectivity, three unique pairs of mass-labeled, complementary peptoids sequences (shown in Fig. 3a) were initially each subjected to the stepwise, dissociation/extraction/annealing assembly process described above. The mass spectra of each reaction mixture demonstrate the successful assembly of the target 10101 × 01010, 11100 × 00011, and 11111 × 00000 molecular ladders from their respective precursor strands (see Fig. 3b, bottom three black, red, and blue spectra, respectively); for comparison, mass spectra of the 00111 × 11000 and 11111 × 00000 molecular ladder reaction mixtures using the previously determined, single-step reaction conditions are shown in Supplementary Fig. 9. Notably, assembly of the precursor strands in either a parallel or anti-parallel orientation can afford in-registry molecular ladders for both 10101 × 01010 and 11111 × 00000 systems; however, the in-registry 00111 × 11000 ladder product can only be achieved through parallel-oriented strands. Whereas hybridization of 11111 and 00000 homo-oligomers achieves registry under either reaction conditions (Supplementary Fig. 9a), the 00111 × 11000 system results in poor hybridization selectivity under the direct deprotection and assembly reaction conditions (Supplementary Fig. 9b, bottom black spectrum), while the dissociation/extraction/annealing process selectively affords the in-registry, five-rung molecular ladder resulting from the hybridization of the 00111 strand with its 11000 complement in a parallel orientation (Supplementary Fig. 9b, top red spectrum).

Concurrent hybridization selectivity for these three sets of complementary peptoid pairs was subsequently examined by allowing equimolar amounts of the six oligomeric sequences (i.e., Fig. 3a) to react simultaneously in a single pot reaction mixture. Here, excluding out-of-registry and multimeric reaction products, this system of six individual strands could potentially yield 19 unique duplexes (as the 11111 and 00000 sequences cannot self-hybridize); nevertheless, application of the dissociation/extraction/annealing assembly process enabled each strand to preferentially hybridize with its residue sequence complement, resulting in the selective assembly of three in-registry molecular ladder products, including 10101 × 01010, parallel-oriented 00111 × 11000, and 11111 × 00000 (Fig. 3b, second from top green spectrum). In contrast, the single-step, deprotection and direct assembly reaction conditions again impeded hybridization selectivity of this system, yielding a non-equilibrium mixture of multiple, unidentified dimeric species (Fig. 3b, top black spectrum), highlighting the extraordinary capacity for the dissociation/extraction/annealing process to alleviate the kinetic trapping that commonly accompanies dynamic covalent assembly (Supplementary Fig. 10). The full range of this spectrum (*m*/*z* of 1000–10,000, see Supplementary Fig. 11) again revealed small peaks attributable to single-stranded species, including both unreacted strands and those with either one or two intramolecular imine bonds, while multimeric complexes at higher molecular weights were not observed. Moreover, the concurrent assembly of 10101, 01010, 00111, and 11000 sequences exclusively affords 10101 × 01010 and parallel-oriented 00111 × 11000 ladders rather than the non-complementary cross-species 10101 × 11000 and 01010 × 00111, both of which could potentially form five imine bonds, confirming that pendant group sequence dictates the hybridization selectivity rather than simply maximizing the number of interstrand imine bonds, and strongly suggesting an unconvoluted, ladder-like conformation.

### Information storage and retrieval

**Fig. 4:**
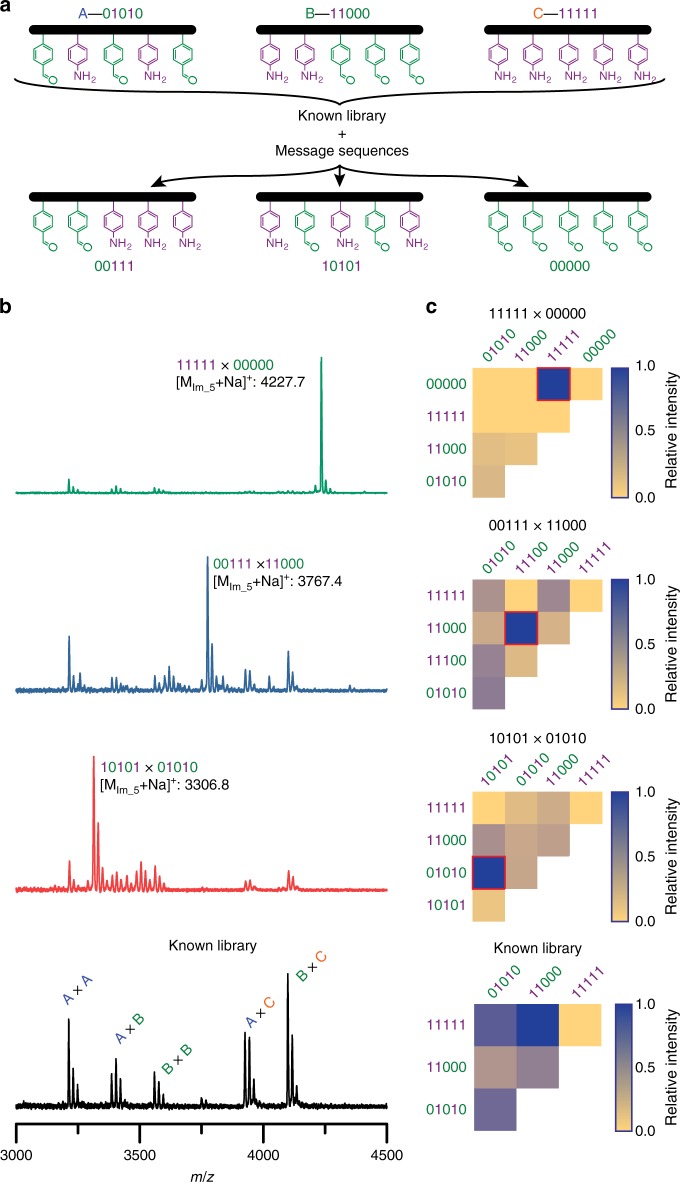
Dynamic covalent information storage and retrieval. **a** Schematic of a known library composed of three unique, mass-labeled oligomeric sequences A (01010), B (11000), and C (11111), and individual messages sequences used to challenge the library. **b** MALDI mass spectrum of the known sequence library treated to the dissociation/extraction/annealing assembly process in the absence of a message strand (black, bottom), resulting in non-specific binding among the library strands, yielding all five possible sets of dimeric ladder species with varying numbers of rungs. Introduction of a message sequence to the known library and treatment of the mixture to the multi-step assembly process yielded MALDI mass spectra with dominant peaks attributable to the in-registry hybridization product of the message strand with its library complement (10101 message, second from bottom, red; 00111 message, second from top, blue; 00000 message, top, green). **c** Hybridization specificity of message strands with their library complements assessed by normalized MALDI mass spectrum intensities. Possible dimeric ladder species are shown; red boxes indicate expected hybrids between message and complementary library strands. Peaks at multiples of +18*m*/*z* values are attributable to ladders species with fewer rungs.

Inspired by the facile nucleic acid sequence identification provided by contemporary DNA microarray technology^41,42^ and the hybridization selectivity of the encoded sequences demonstrated above, we were motivated to explore the versatility of this model system as a potential information retrieval mechanism. Here, messages in the form of binary-encoded peptoid sequences are challenged against a known library of unpaired, mass-labeled oligomers. Selective hybridization of a message peptoid with its complementary sequence should afford a molecular ladder with a pre-determined mass such that the message can be identified through mass spectrometry on the reaction mixture. To validate this concept, the mass-labeled, acetal-protected peptoid oligomers 01010, 11000, and 11111 were used in equimolar amounts as a known library of potential complementary sequences (Fig. 4a). Initially, treatment of this library to the dissociation/extraction/annealing assembly process in the absence of a message strand resulted in non-specific binding among the library strands, yielding five sets of dimeric ladder species with varying numbers of rungs (see Fig. 4b, bottom black spectrum). Upon addition of a sub-stoichiometric amount of a 00111 message strand to a fresh sequence library and application of the dissociation/extraction/annealing process, characterization of the reaction mixture by mass spectrometry revealed a dominant new peak attributable to a selectively hybridized, in-registry ladder composed of the message oligomer and its complementary strand from the known library (Fig. 4b, second from top blue spectrum), successfully demonstrating the capacity of this system to convey information despite the presence of competitive binding species in excess. Similar results were obtained for 01010 and 00000 message strands when challenged against the oligomer library (see Fig. 4b, second from bottom red and top green spectra, respectively). To determine the detection limit for this message identification approach, varying sub-stoichiometric amounts of a 10101 message strand were added to the known sequence library described above (see Supplementary Fig. 12a) and, after treatment with the dissociation/extraction/annealing process, the mixtures were characterized again by mass spectrometry. Whereas peaks attributable to the 10101 × 01010 ladder product were readily apparent in the mass spectrum at 0.2 equivalents of the message strand, addition of 0.75 equivalents of the message were necessary for a peak attributable the in-registry ladder product generated from the message strand and its complementary sequence to become dominant in the mass spectrum (Supplementary Fig. 12b and Supplementary Table 2).

The ability to direct the self-assembly of oligomeric strands based on their residue sequence and mediated by dynamic covalent interactions as demonstrated here is a crucial step towards the fabrication of complex, unimolecular constructs from modest, synthetically accessible precursors. By employing a step-wise process technique involving Sc(OTf)_3_ as a multi-role reagent to deprotect acetal-protected aldehyde groups, affect the equilibrium of the amine/aldehyde condensation reaction, and catalyze the rearrangement of inter-strand imine bonds, the kinetic trapping of non-equilibrium species that often prevails in dynamic covalent assembly systems is mitigated, allowing the system to converge towards thermodynamic equilibrium through sequence-selective assembly of molecular ladders from oligo(peptoid)s. By employing a dissociation/annealing assembly process, other dynamic covalent interactions could be utilized to mediate selective sequence assembly (e.g., boronic acid/diol condensation, Diels–Alder cycloaddition, etc.), similarly mimicking that of nucleic acids. Although this study involved molecular ladders bearing covalent rungs, this multi-step approach to dynamic covalent assembly process may also be useful for other applications in which the alleviation or elimination of kinetic trapping is critical. Specifically, this process will provide significantly improved synthetic access to robust, complex covalent nanostructures, such as molecular cages and crystalline, porous polymer networks.

## Methods

### General experimental procedures

Unless otherwise stated, chemicals and reagents were obtained from commercial sources and used as received. ^1^H NMR spectra were collected using Varian MR400 and Varian Inova 500 spectrometers. DOSY NMR spectra were collected using a Bruker 400 MHz NMR spectrometer. Chemical shifts were measured in *δ* (ppm) relative to residual solvent signals as internal standards (CDCl_3_—7.24 for ^1^H; CD_3_CN—1.94 for ^1^H). Matrix-assisted laser desorption/ionization (MALDI) mass spectra were recorded using a Bruker Autoflex mass spectrometer, whereas electrospray ionization (ESI) mass spectra were recorded using an Agilent Q-TOF 1200 series spectrometer. MALDI analyses were performed in reflectron positive ion mode using 2-(4-hydroxyphenylazo)benzoic acid (HABA) as the matrix, where 2 μL of a solution of the sample (1 mM) was mixed with 6 μL of a mixture of 10 mg matrix in 200 μL acetonitrile, spotted on a MALDI sample plate (Bruker), and allowed to air dry. Reverse phase high performance liquid chromatography (RP-HPLC) was performed using a Shimadzu LC-6AD HPLC pump, equipped with a Shimadzu FRC 70A fraction collector, using analytical and preparative reversed phase Phenomenex Luna C18(2) columns with a linear gradient of water and acetonitrile as the eluent at 30 °C, and monitored with a Shimadzu Prominence UV/vis detector at 214 nm. GPC was performed using a Shimadzu LC-20AD HPLC pump equipped with three Phenogel GPC/SEC columns (length 300 mm × diameter 7.8 mm, pore sizes of 500, 100, and 50 Å) in series, 94:4:2 (v/v/v) CHCl_3_:MeOH:Et_3_N as the eluent at room temperature, and monitored with a Shimadzu Prominence UV/vis detector at 313 nm. GPC calibration was performed using low dispersity polystyrene standards (low molecular weight Readycal Set, Fluka).

### Monomer synthesis

Primary amine monomers were purchased or synthesized according to published approaches^34,35^ and can be seen in Supplementary Table 1.

Npal: Npal was synthesized by addition of 42.2 mL ethylene glycol, 0.02 g toluene *p*-sulfonic acid, and 25 g of 4-cyanobenzaldehyde to 200 mL of toluene. After refluxing overnight with a Dean–Stark trap to remove water generated during the reaction, the reaction mixture was cooled to room temperature and 40 mL of 5% NaHCO_3_ aqueous solution was added. The organic layer was extracted, washed with deionized water three times, and evaporated to yield 4-(1,3-dioxacyclopent-2-yl)benzonitrile. 10 g of 4-(1,3-dioxacyclopent-2-yl)benzonitrile in 100 mL dry diethyl ether was added dropwise into 4.33 g of LiAlH_4_ in 100 mL dry diethyl ether under nitrogen. The reaction mixture was stirred for 4 h at 0 °C and 12 h at room temperature, then quenched by 95% ethanol, and further quenched by 50% ethanol in water. The ether supernatant was dried under reduced pressure to yield Npal.

Npam: 4.9 g allyl chloroformate in 150 mL 1,4-dioxane was added into a solution of 4-(2-aminoethyl)aniline (5 g) in 150 mL 10% aq. acetic acid. The reaction mixture was stirred overnight at room temperature and then diluted with water prior to washing with diethyl ether. The aqueous layer was adjusted to pH 14 by 2 M NaOH (aq) and was extracted by diethyl ether. The organic fraction was evaporated to yield Npam.

Neee: 50 mL of 6 M NaOH was added to 20 g of diethylene glycol monoethyl ether and 50 mL of tetrahydrofuran (THF) cooled to 0 °C, followed by dropwise addition of 54 g tosyl chloride in 80 mL THF under N_2_. After stirring for 1 h at 0 °C, the reaction mixture was left for an additional hour at room temperature. The mixture was extracted with diethyl ether and the organic layer washed with 1 M NaOH and water before evaporating under vacuum to yield 2-(2-ethoxyethoxy)ethyl tosylate. 40 g of 2-(2-ethoxyethoxy)ethyl tosylate and 250 mL of DMF were charged to a 500 mL flask under N_2_. 31.5 g of NaN_3_ was added to the reaction mixture which was then stirred at 60 °C for 36 h before cooling to room temperature. The reaction mixture was extracted with diethyl ether and washed with water before evaporating under vacuum to produce 2-(2-ethoxyethoxy)ethyl azide. 2-(2-Ethoxyethoxy)ethyl azide (20 g) in 160 mL THF and 40 g triphenylphosphine were charged to a 250 mL flask under nitrogen. Upon stirring overnight, the reaction mixture was quenched with 220 mL water and allowed to stir for another day. The resulting solution was washed with toluene and dichloromethane, and evaporated under vacuum to yield Neee.

Ndab: To a 20 mL vial equipped with magnetic stirrer, 500 mg of DABCYL succinimide ester and 10 mL (excess) of distilled ethylene diamine were added. The reaction was allowed to stir for 12 h and then excess ethylene diamine was removed by rotary evaporation. The product was extracted by dichloromethane and then washed with distilled water to remove excess amine and succinimide. The organic layer was dried over Na_2_SO_4_, filtered and solvent removed by rotary evaporation to yield Ndab.

### Oligo(peptoid) synthesis

Peptoids were synthesized via a sub-monomer approach to solid phase synthesis using Fmoc-Photolabile SS (0.1 mmol scale, 100–200 mesh, 1% DVB, Advanced ChemTech, Kentucky) as the resin. Syntheses were performed in an automated microwave synthesizer (Liberty Blue, CEM Corporation, North Carolina) adapted for peptoid synthesis. Resin was swelled at room temperature for 5 min with DMF before deprotection with 20% 4-methylpiperidine in DMF (v/v) for 30 s at 75 °C and 90 s at 90 °C. Subsequently, alternating additions of 1 mL of 1 M bromoacetic acid (coupled with 1 mL of 1 M *N*,*N*′-diisopropylcarbodiimide (DIC)) and 2.5 mL of 0.5 M primary amine monomer were repeated until desired sequence length was achieved. Primary amine monomers consisted of Neee as the principal spacer, an allyloxycarbonyl- (Alloc) protected amine (Npam protected, Nam unprotected), an ethylene acetal-protected aldehyde (Npal protected, Nal unprotected), and Nme as a mass label (Supplementary Table 1). The *N*-terminal of the oligomers were capped with 1 M acetic anhydride, activated by DIC. Fluorescein-functionalized oligomers were synthesized by omitting the acetic anhydride capping step and instead of introducing a final coupling step by adding 1 mL of a 0.4 M 5,6-carboxyfluoroscein solution in DMF in addition to 1 mL 0.6 M HCTU in DMF and 1.5 mL of 0.8 M *N*,*N*-diisopropylethylamine in DMF.

Oligo(peptoids) were subjected on-resin to 0.1 equivalents of tetrakis(triphenylphosphine) palladium(0) and 25 equivalents of phenylsilane per Alloc group in dry DCM for one hour to deprotect the Alloc-amines. The deprotection solution was filtered off, and peptoids were introduced to a fresh deprotection solution for another hour. Upon filtration of the second solution, the peptoids on-resin were submerged in 5 mL DMF and cleaved for 36 h under irradiation at ~25 mW cm^−2^ with 405 nm. Resin was separated from liberated peptoids with the use of a syringe filter. Solvent was removed under vacuum, and the oligomers were reconstituted in acetonitrile and further purified in preparative HPLC using a linear gradient of acetonitrile (MeCN) and water: (1) 30% MeCN, 0.1–2.1 min; (2) 30–95% MeCN, 2.1–16.1 min; (3) 95% MeCN, 16.1–23.1 min; (4) 95% MeCN, 23.1–26.1 min. Purified peptoids were lyophilized to yield off-white powder.

### Peptoid hybridization

Conventional single-step method: A vial was charged with 20 µL of 10 mM solutions of each of the desired oligo(peptoid) sequences in acetonitrile, 0.2 eq. of Sc(OTf)_3_ per potential imine bond from a 10 mM stock solution, and sufficient solvent to afford 200 µL total of a 2% (v/v) of water/acetonitrile solution. The mixture stirred gently for 2 h at 70 °C.

Dissociation/extraction/annealing method: Dissociation of peptoid sequences was achieved by adding a rare-earth metal triflate (either 1.5 eq. of Sc(OTf)_3_, Yb(OTf)_3_, or Lu(OTf)_3_, or 3.0 eq. of Y(OTf)_3_ or Sm(OTf)_3_ per potential imine bond) from a 10 mM stock solution to a 200 µL 2% (v/v) of water/acetonitrile solution containing 0.2 µmol of each desired oligo(peptoid) sequence and heating at 70 °C. After heating for 2 h, 200 µL of chloroform and 2 mL of water were added to the vial, followed by gentle shaking. The mixture was allowed to stand and, upon complete phase separation^30^, the organic layer was extracted and stirred in a new vial at 70 °C for 2 h for oligomer annealing.

Sequence identification method: Sequence identification hybridizations were similarly performed by adding 15 µL of a 10 mM solution of ‘message’ peptoid to a vial containing 200 µL of a 2% (v/v) of water/acetonitrile solution, 0.2 µmol of each peptoid strand constituting the known library (01010, 11000, and 11111) and 0.225 µmol of Sc(OTf)_3_. As before, this solution was heated at 70 °C for 2 h, then diluted with 200 µL of chloroform. After heating, 2 mL of water was added and the solution was gently shaken. Upon isolation of the chloroform layer, annealing was carried out at 70 °C for 2 h.

### Förster resonance energy transfer

Quantification of hybridization selectivity was achieved via the dissociation/extraction/annealing of 1 mM hybridization mixtures initially consisting of distinct combinations of 01010-FAM, 10101, 10001, 10101-DABCYL, and 10001-DABCYL strands in stoichiometric ratios and 1.5 eq. Sc(OTf)_3_ in 200 µL of a 2% (v/v) of water/acetonitrile solution. After annealing at 70 °C for 6 h and room temperature for one day, 200 µL of a pH 9 bicarbonate–carbonate buffer solution was added to each hybridization mixture. The hybridized solutions were further diluted to 1.2 mL with acetonitrile, then the fluorescence of the resultant solutions was recorded with a Horiba Quanta Master fluorimeter by exciting at 495 nm and measuring the fluorescence intensity at 525 nm. Triplicates of each solution were tested and normalized to a control mixture of 01010-FAM that was subject to the same dissociation/extraction/annealing process. Results are reported with standard error.

### Inductively coupled plasma mass spectrometry

Post-dissociation/extraction solutions of hybridized 11111 × 00000, 00111 × 11000, or 10101 × 01010 (initially 1 mM in 200 µL 2% (v/v) of water/acetonitrile and either 1.5 μmol of Sc(OTf)_3_, Yb(OTf)_3_, or Lu(OTf)_3_, or 3.0 μmol of Y(OTf)_3_ or Sm(OTf)_3_) in CHCl_3_ were dried under a steady stream of nitrogen and reconstituted in 1 mL of 2% nitric acid (aq). Samples were diluted 4 × 10^6^-fold with Milli-Q water and the residual scandium concentrations were determined by ICP-MS using a Perkin-Elmer NexION 2000. Experiments on each solution were performed in triplicate and results are reported with standard errors.

## Supplementary information

Supplementary Information

## Data Availability

The data that support the findings of this study are available from the corresponding author upon reasonable request.
