# Supplementary material for: Sequence-selective dynamic covalent assembly of information-bearing oligomers
Source: Nat Commun. 2020 Feb 7;11:784. doi: 10.1038/s41467-020-14607-3 (PMC7005811; doi:10.1038/s41467-020-14607-3)
Supplement: Supplementary file 1 — Supplementary Information [file 41467_2020_14607_MOESM1_ESM.pdf]

# Supplementary Information for

# Sequence-selective Dynamic Covalent Assembly of Information-bearing Oligomers

Samuel C. Leguizamon and Timothy F. Scott

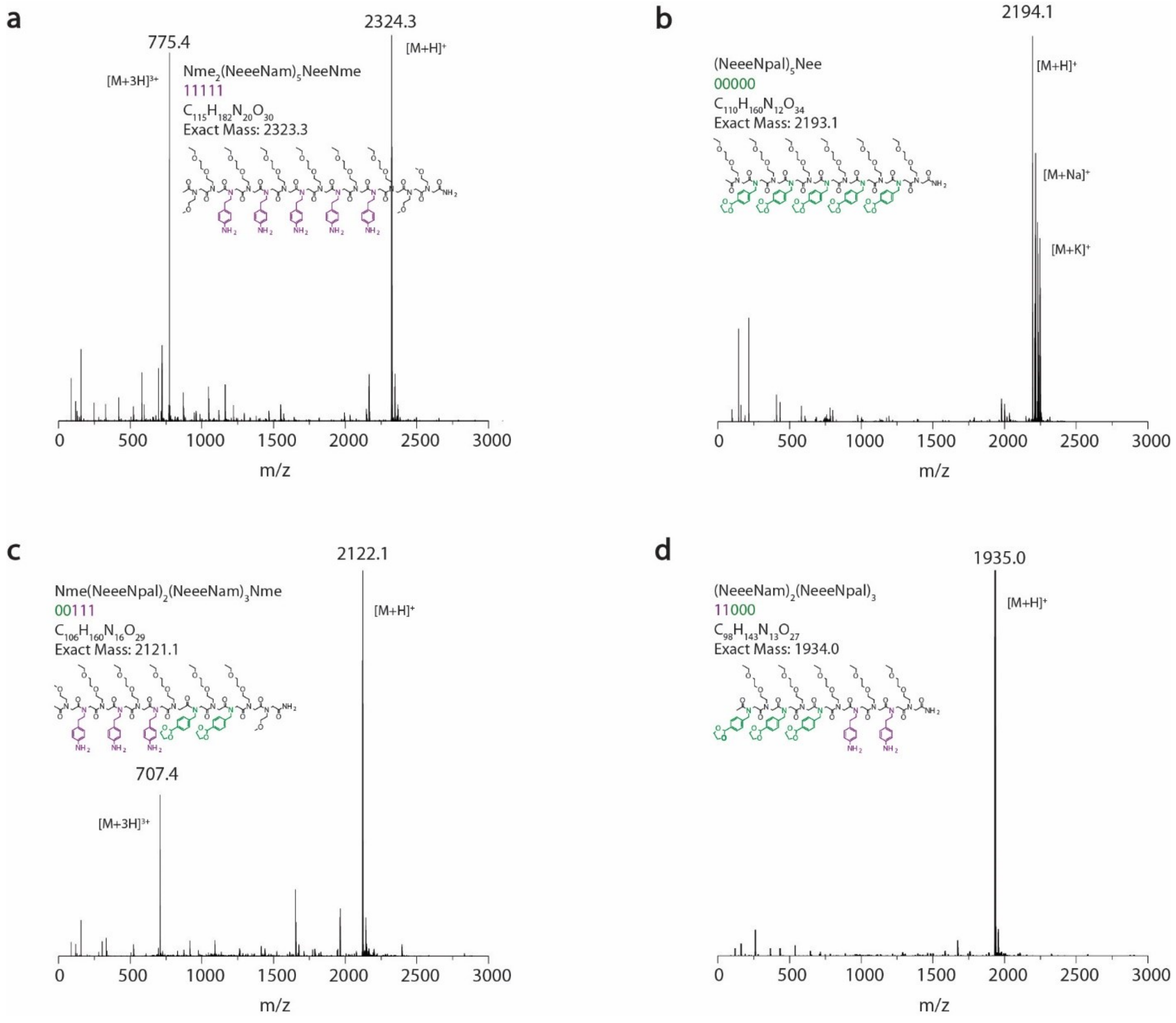

a
775.4
2324.3
[M+3H]3+
[M+H]+
Nme2(NeeeNam)5NeeNme
11111
C115H182N20O30
Exact Mass: 2323.3
m/z
b
2194.1
(NeeeNpal)5Nee
00000
C110H160N12O34
Exact Mass: 2193.1
[M+H]+
[M+Na]+
[M+K]+
m/z
c
2122.1
Nme(NeeeNpal)2(NeeeNam)3Nme
00111
C106H160N16O29
Exact Mass: 2121.1
[M+H]+
707.4
[M+3H]3+
m/z
d
1935.0
(NeeeNam)2(NeeeNpal)3
11000
C98H143N13O27
Exact Mass: 1934.0
[M+H]+
m/z

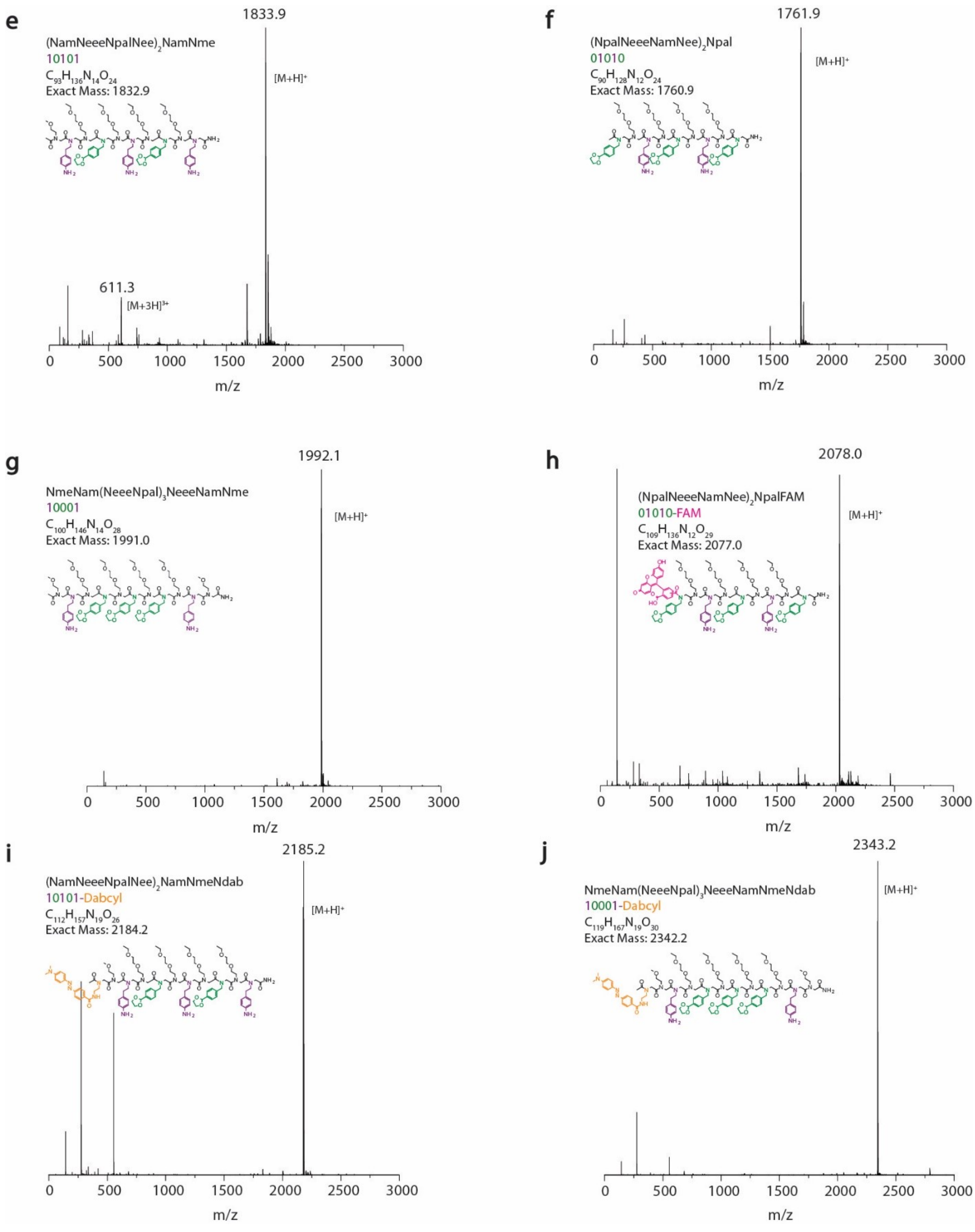


**Supplementary Figure 1** ESI mass spectra of purified peptoid sequences;
**a,** Nme$_2$(NeeeNam)$_5$NeeNme; **b,** (NeeeNpal)$_5$Nee; **c,** Nme(NeeeNpal)$_2$(NeeeNam)$_3$Nme;
**d,** (NeeeNam)$_2$(NeeeNpal)$_3$; **e,** (NamNeeeNpalNee)$_2$NamNme; **f,** (NpalNeeeNamNee)$_2$Npal;
**g,** NmeNam(NeeeNpal)$_3$NeeeNamNme; **h,** (NpalNeeeNamNee)$_2$NpalFAM;
**i,** (NamNeeeNpalNee)$_2$NamNmeNdab; **j,** NmeNam(NeeeNpal)$_3$NeeeNamNmeNdab.

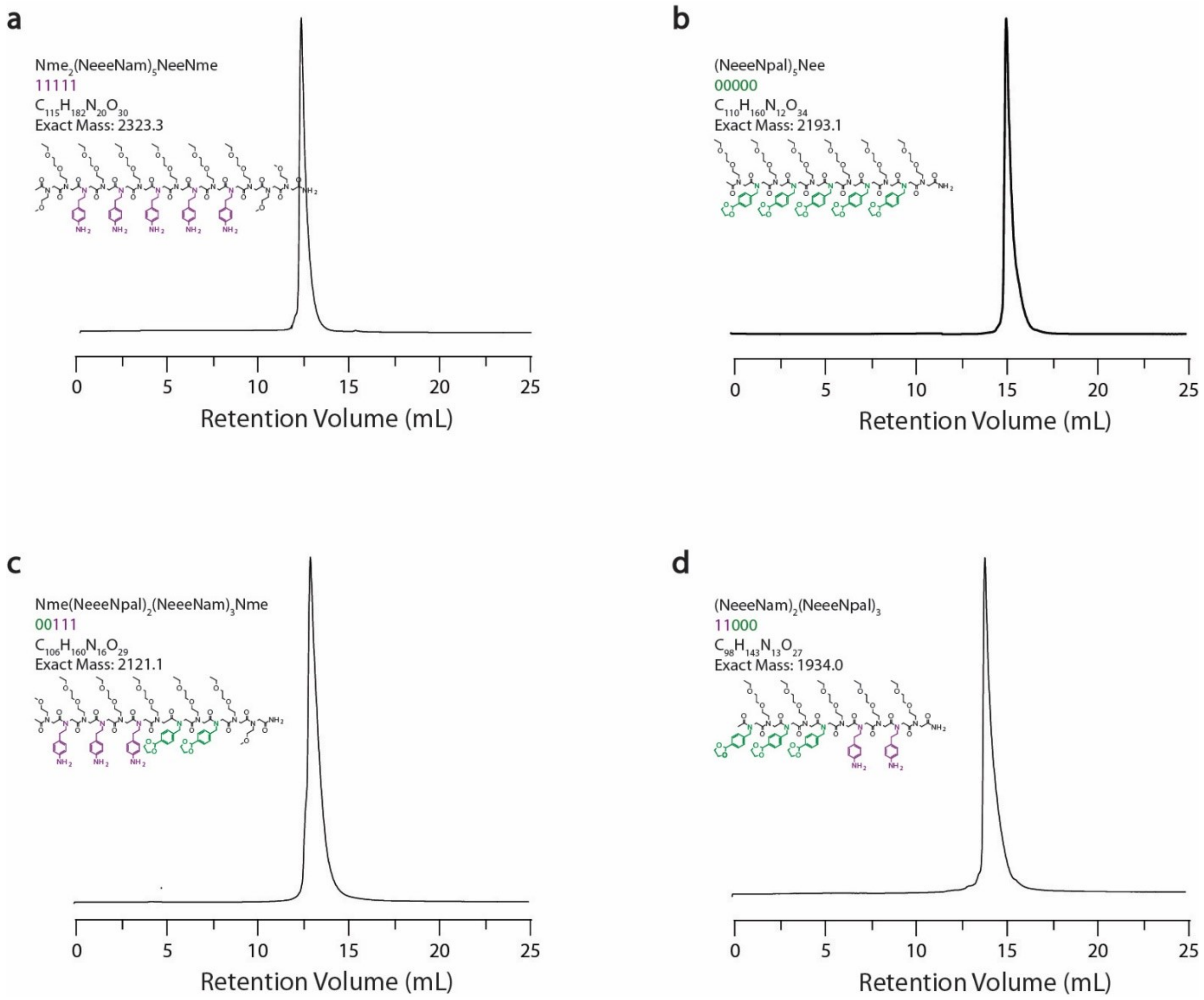
a
Nme2(NeeeNam)5NeeNme
11111
C115H182N20O30
Exact Mass: 2323.3
0 5 10 15 20 25
Retention Volume (mL)
b
(NeeeNpal)5Nee
00000
C110H160N12O34
Exact Mass: 2193.1
0 5 10 15 20 25
Retention Volume (mL)
c
Nme(NeeeNpal)2(NeeeNam)3Nme
00111
C106H160N16O29
Exact Mass: 2121.1
0 5 10 15 20 25
Retention Volume (mL)
d
(NeeeNam)2(NeeeNpal)3
11000
C98H143N13O27
Exact Mass: 1934.0
0 5 10 15 20 25
Retention Volume (mL)

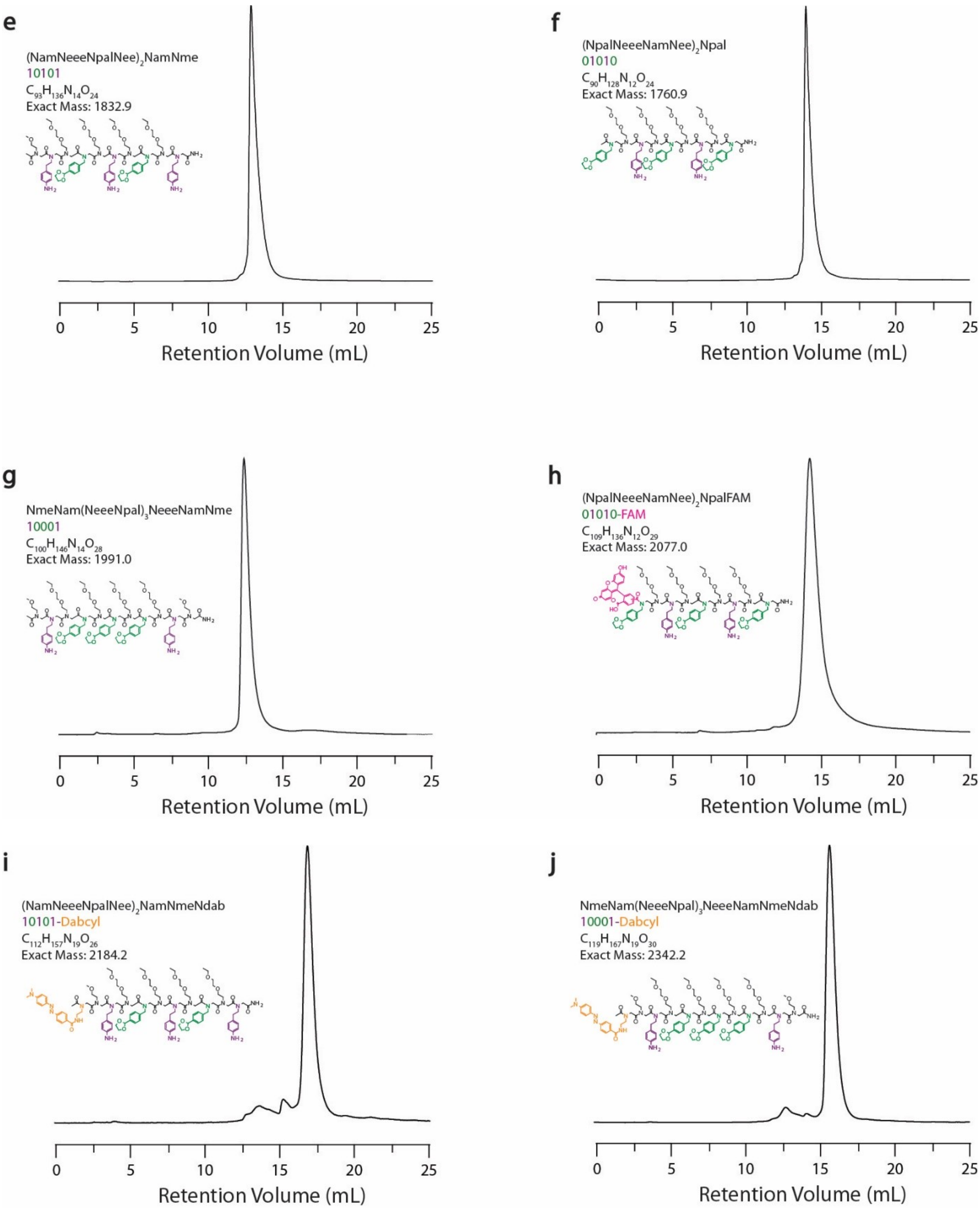


**Supplementary Figure 2 a-j,** RP-HPLC traces of purified peptoid sequences.
**a,** $Nme_2(NeeeNam)_5NeeNme$; **b,** $(NeeeNpal)_5Nee$; **c,** $Nme(NeeeNpal)_2(NeeeNam)_3Nme$; **d,** $(NeeeNam)_2(NeeeNpal)_3$; **e,** $(NamNeeeNpalNee)_2NamNme$; **f,** $(NpalNeeeNamNee)_2Npal$; **g,** $NmeNam(NeeeNpal)_3NeeeNamNme$; **h,** $(NpalNeeeNamNee)_2NpalFAM$; **i,** $(NamNeeeNpalNee)_2NamNmeNdab$; **j,** $NmeNam(NeeeNpal)_3NeeeNamNmeNdab$.

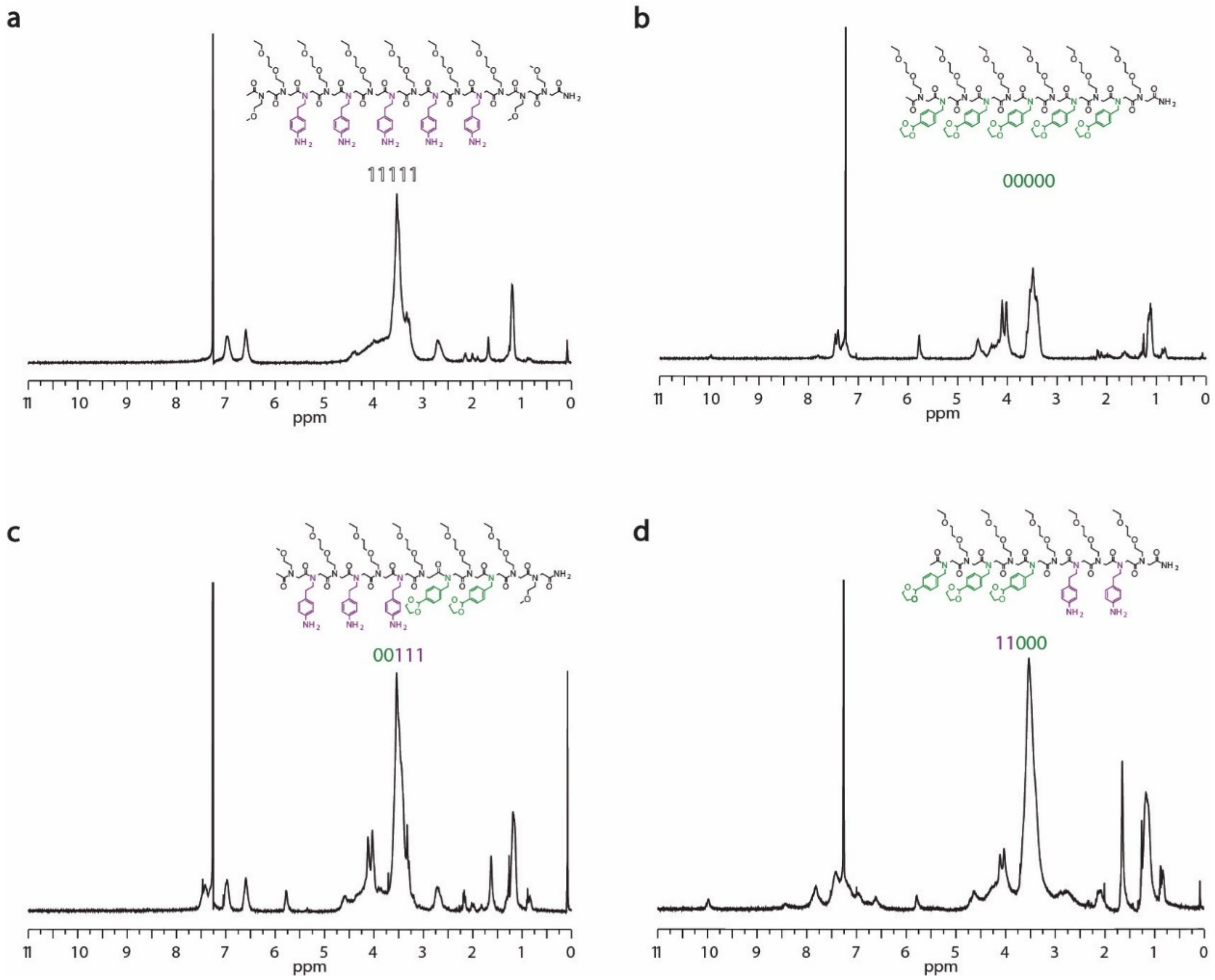
a
11111
b
00000
c
00111
d
11000
ppm
ppm
ppm
ppm

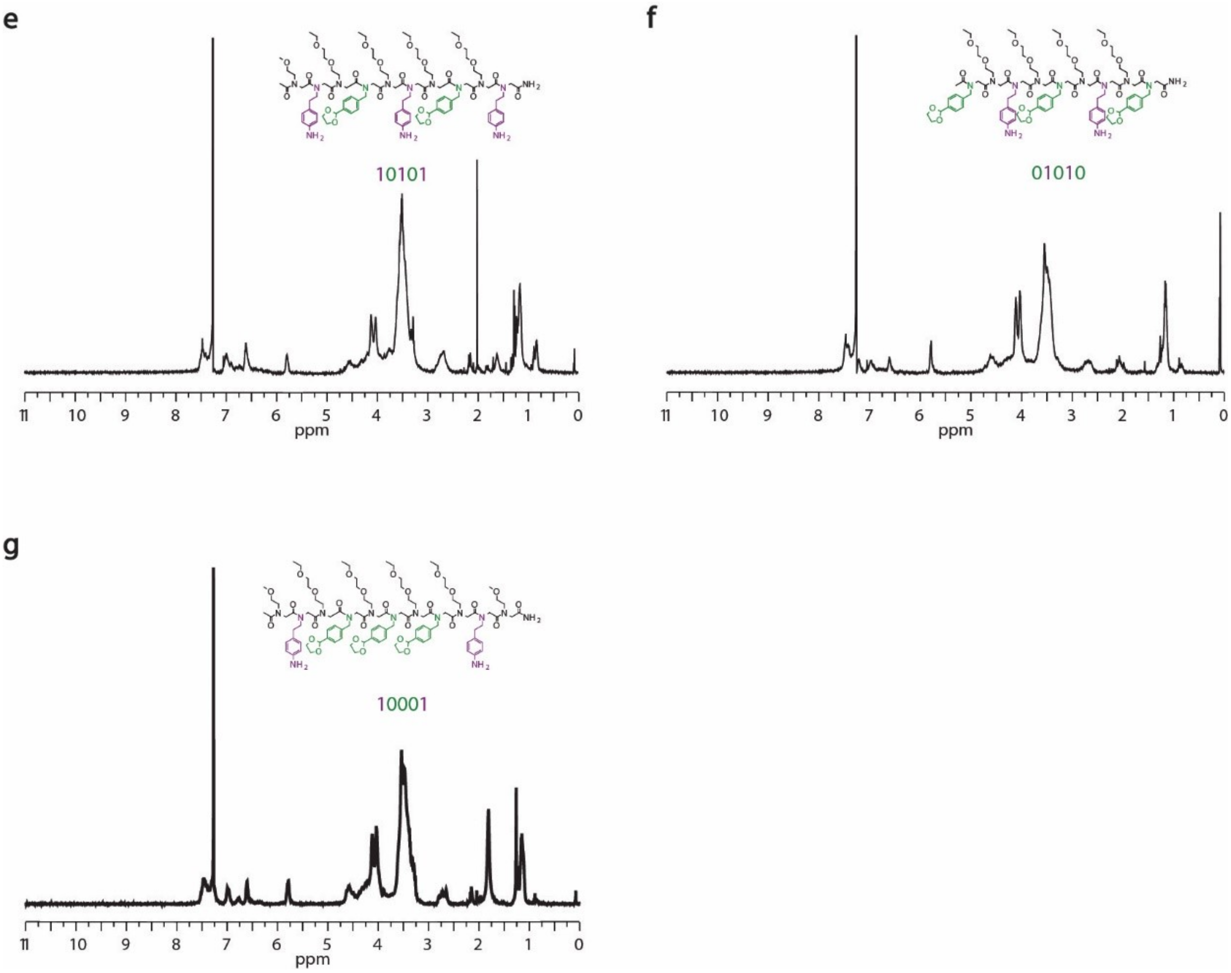


**Supplementary Figure 3** $^1$H NMR spectrum (500 MHz, $CDCl_3$) of purified peptoid sequences.

a. **11111**: δ (ppm): 6.85-7.05 (m, Ar), 6.45-6.70 (m, Ar), 3.00-4.53 (br, -N-C$H_2$-CO-, -O-C$H_2$-C$H_2$-O-, Ar-$CH_2$-C$H_2$-N-, -O-C$H_3$, -N-C$H_2$-C$H_2$-O-, -OC$H_2$-$CH_3$), 2.50-2.80 (br, Ar-C$H_2$-$CH_2$-N-), 1.58-1.73 (m, -CO-C$H_3$), 1.00-1.21 (m, -O-$CH_2$-C$H_3$)

b. **00000:** δ (ppm): 9.95-10.07 (m, Ar-C*H*O), 7.60-8.00 (m, Ar), 7.31-7.58(m, Ar), 5.76-5.80 (m, -Ar-C*H*-$O_2$-), 3.13-4.80 (m, Ar-C$H_2$-N-, -N-C$H_2$-C$H_2$-O-, -N-C$H_2$-CO-, -O-C$H_2$-C$H_2$-O-, -O-C$H_2$-$CH_3$, -(O-C$H_2$)$_2$-, Ar-C$H_2$-$CH_2$-N-), 2.06-2.18 (m, -CO-C$H_3$), 1.05-1.20 (m, -O-$CH_2$-C$H_3$)

c. **00111:** δ (ppm): 7.33-7.54 (m, Ar), 7.25-7.35 (m, Ar), 6.90-7.04 (m, Ar), 6.51-6.66 (m, Ar), 5.64-5.88 (m, -Ar-C*H*-$O_2$-), 3.13-4.86 (br, Ar-C$H_2$-N-, -O-C$H_2$-C$H_2$-O-, Ar-C$H_2$-$CH_2$-N-, -N-C$H_2$-C$H_2$-O-, -O-C$H_3$, -N-C$H_2$-CO-,-O-C$H_2$-$CH_3$, -(O-C$H_2$)$_2$-,), 2.50-2.84 (br, Ar-$CH_2$-C$H_2$-N-), 1.51-1.70 (m, -CO-C$H_3$), 0.99-1.39 (-O-$CH_2$-C$H_3$)

d. **11000:** δ (ppm): 9.96-10.01 (m, Ar-C*H*O), 7.63-7.97 (m, Ar), 7.33-7.54 (m, Ar), 6.85-7.02 (m, Ar), 6.46-6.70 (m, Ar), 5.68-5.85 (m, -Ar-C*H*-$O_2$-), 3.05-4.81 (br, Ar-C$H_2$-N-, -O-C$H_2$-C$H_2$-O-, Ar-C$H_2$-$CH_2$-N-, -N-C$H_2$-C$H_2$-O-, -O-C$H_3$, -N-C$H_2$-CO-,-O-C$H_2$-$CH_3$, -(O-C$H_2$)$_2$-), 2.52-2.99 (br, Ar-$CH_2$-C$H_2$-N-), 1.50-1.72 (m, -CO-C$H_3$), 0.96-1.31 (m, -O-$CH_2$-C$H_3$)

e. **10101:** δ (ppm): 7.36-7.52 (m, Ar), 7.24-7.34 (m, Ar), 6.88-7.08 (m, Ar), 6.49-6.66 (m, Ar), 5.72-5.86 (m, -Ar-C*H*-$O_2$-), 3.17-4.88 (br, Ar-C$H_2$-N-, -O-C$H_2$-C$H_2$-O-, Ar-C$H_2$-$CH_2$-N-, -N-C$H_2$-C$H_2$-O-, -O-C$H_3$, -N-C$H_2$-CO-,-O-C$H_2$-$CH_3$, -(O-C$H_2$)$_2$-), 2.54-2.87 (br, Ar-$CH_2$-C$H_2$-N-), 1.50-1.62 (m, -CO-C$H_3$), 0.99-1.33 (m, -O-$CH_2$-C$H_3$)

f. **01010:** δ (ppm): 7.34-7.53 (m, Ar), 7.10-7.34 (m, Ar), 6.90-7.07 (m, Ar), 6.51-6.66 (m, Ar), 5.66-5.92 (m, -Ar-C$H$-$O_2$-), 3.15-5.18 (br, Ar-C$H_2$-N-, -O-C$H_2$-C$H_2$-O-, Ar-C$H_2$-$CH_2$-N-, -N-C$H_2$-C$H_2$-O-, -O-C$H_3$, -N-C$H_2$-CO-,-O-C$H_2$-$CH_3$, -(O-C$H_2$)$_2$-), 2.51-2.94 (br, Ar-$CH_2$-C$H_2$-N-), 1.50-1.62 (m, -CO-C$H_3$), 0.99-1.35 (m, -O-$CH_2$-C$H_3$)

g. **10001:** δ (ppm): 7.32-7.55 (m, Ar), 7.21-7.34 (m, Ar), 6.91-7.01 (m, Ar), 6.55-6.63 (m, Ar), 5.70-5.83 (m, -Ar-C$H$-$O_2$-), 3.21-4.70 (br, Ar-C$H_2$-N-, -O-C$H_2$-C$H_2$-O-, Ar-C$H_2$-$CH_2$-N-, -N-C$H_2$-C$H_2$-O-, -O-C$H_3$, -N-C$H_2$-CO-,-O-C$H_2$-$CH_3$, -(O-C$H_2$)$_2$-), 2.58-2.82 (br, Ar-$CH_2$-C$H_2$-N-), 1.66-1.90 (m, -CO-C$H_3$), 1.05-1.32 (m, -O-$CH_2$-C$H_3$)

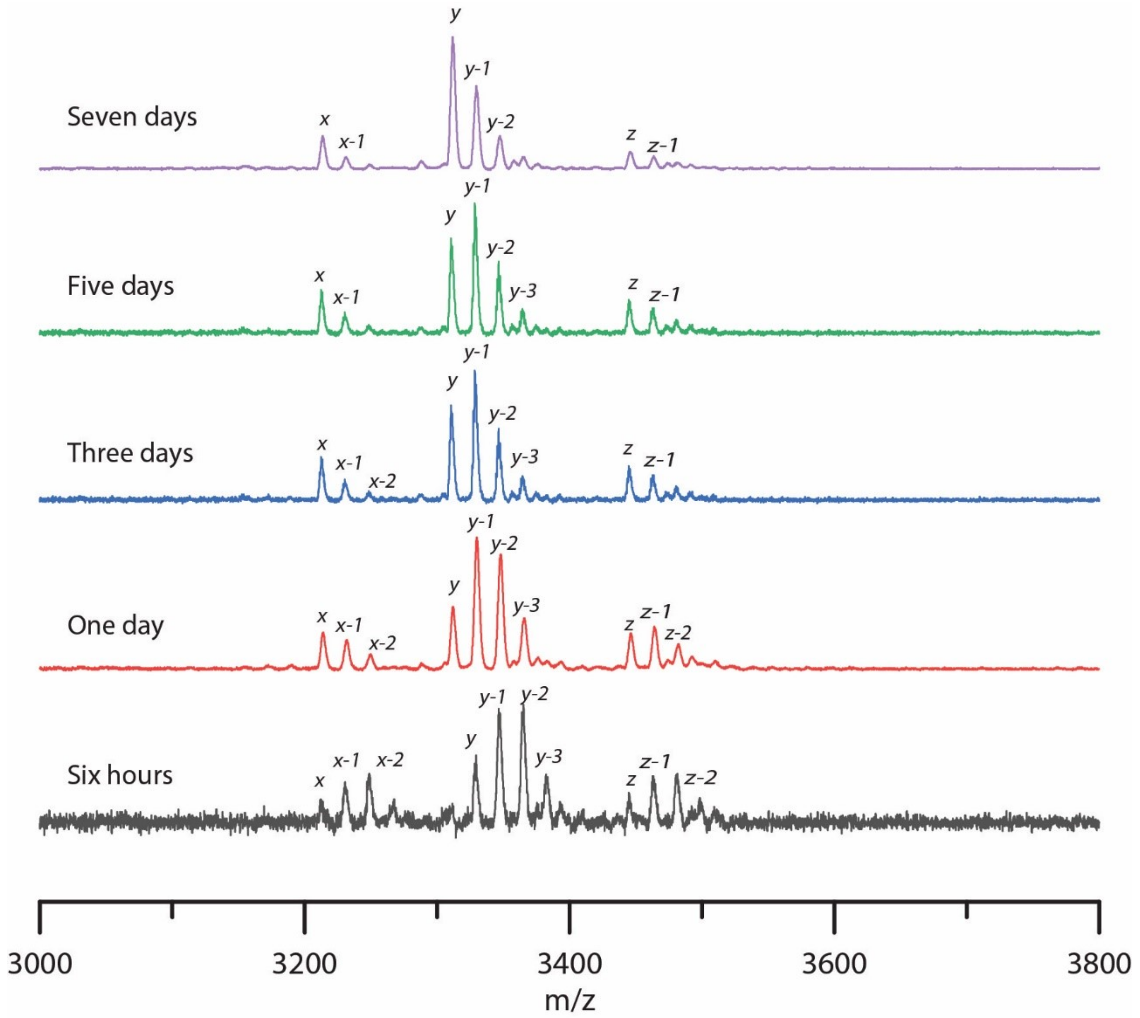


**Supplementary Figure 4** MALDI mass spectra of a 10101×01010 molecular ladder reaction mixture annealed at room temperature over a seven-day period. Expected exact masses: $[M_{01010\times01010}+Na]^+$ ($x$) = 3208.63; $[M_{10101\times01010}+Na]^+$ ($y$) = 3306.71; $[M_{10101\times10101}+Na]^+$ ($z$) = 3440.81. Peaks at multiples of +18 m/z values are attributable to ladders species with fewer rungs (e.g., *y* = in-registry, 5-rung 10101×01010 molecular ladder, *y*-1 = out-of-registry, 4-rung 10101×01010 molecular ladder, etc.).

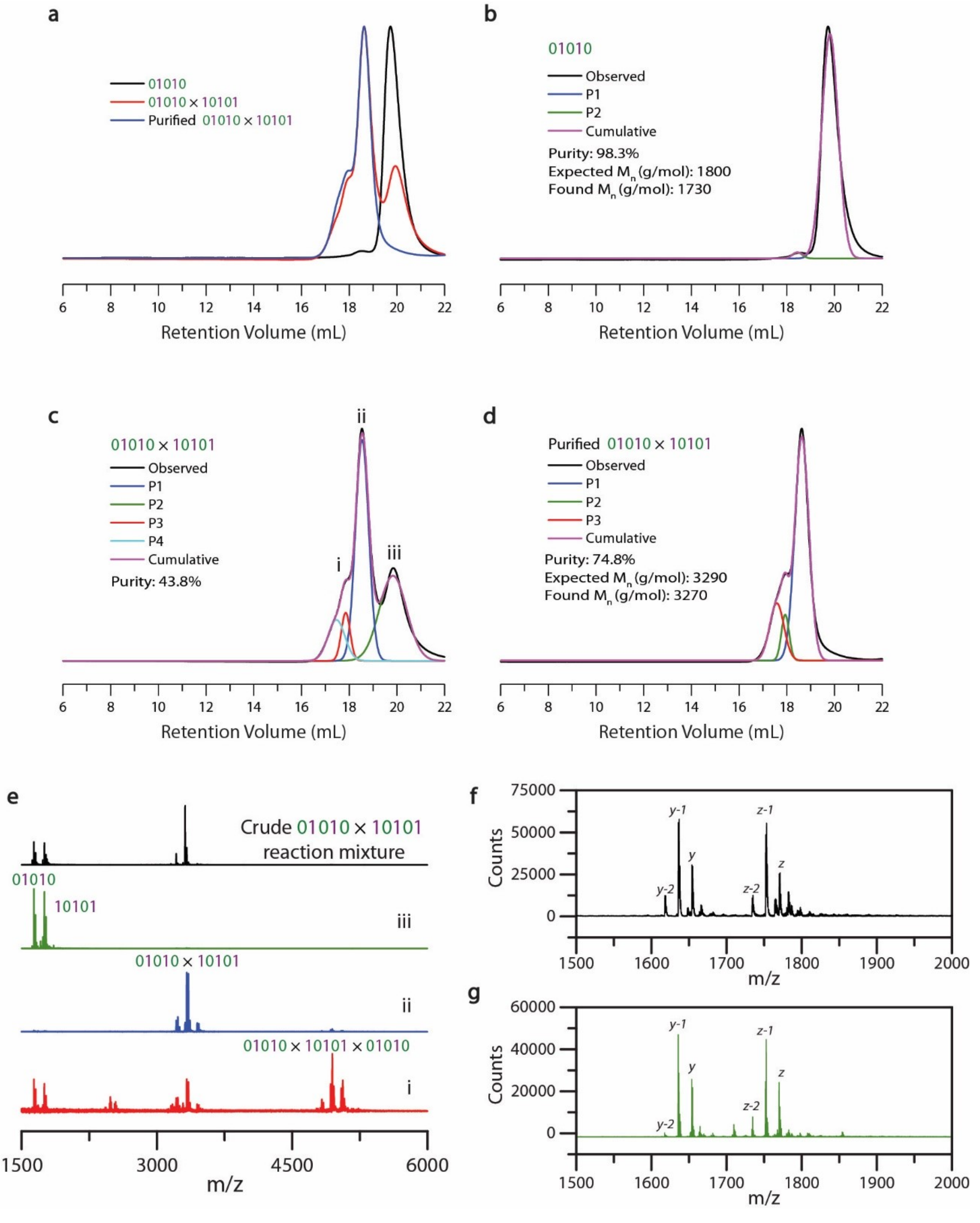


**Supplementary Figure 5 a**, Superimposed GPC traces of an Alloc-protected, inert single strand mixture, a 10101×01010 molecular ladder mixture after performing the dissociation/extraction/annealing method, and a post-purification 10101×01010 molecular ladder. Each trace was deconvoluted (**b**, **c**, and **d**) by fitting Gaussian functions to simulated peaks with OriginPro. Upon subtracting the baseline, each peak over 0.5% of the max intensity was utilized to fit the spectra. **e**, MALDI mass spectra of the different fractions (i, ii, and iii) collected from a 10101×01010 molecular ladder mixture after GPC purification. The peaks in the molecular weight region between 4750 and 5250 correspond to 01010×01010×01010, 01010×01010×10101, and 01010×10101×10101 triple ladder species. MALDI mass spectra expanded to highlight the m/z range expected for single-stranded oligomeric species of **f**, the crude 01010×10101 reaction mixture, and **g**, GPC fraction iii. Expected exact masses:

$[M_{01010}+Na]^+$ ($y$) = 1651.83; $[M_{10101}+Na]^+$ ($z$) = 1767.92. Peaks at multiples of -18 m/z values are attributable to single-stranded species incorporating intramolecular imine bonds (e.g., $y$ = 01010, $y$-1 = 01010 with one intramolecular imine bond, $y$-2 = 01010 with two intramolecular imine bonds, etc.).

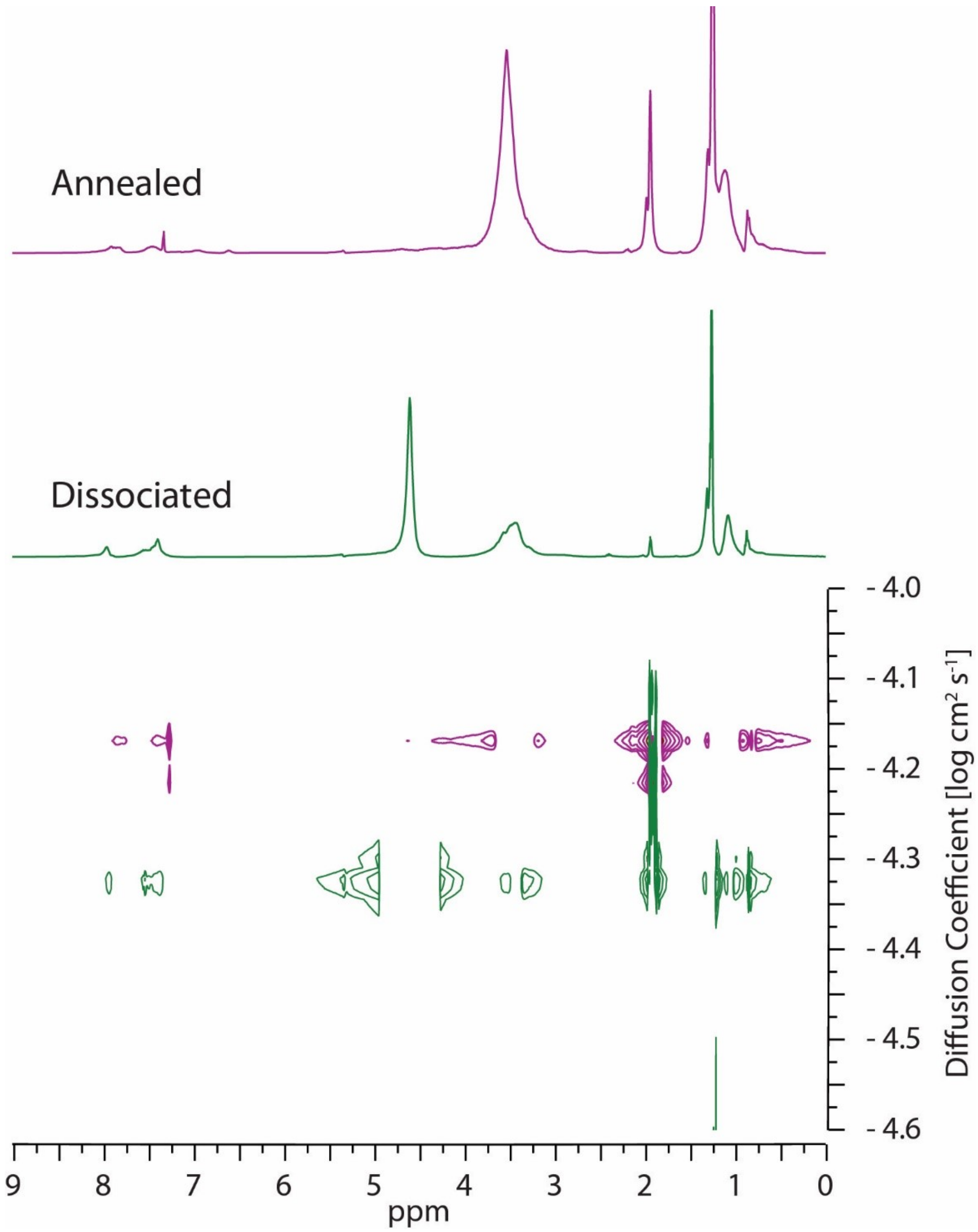


**Supplementary Figure 6** DOSY NMR spectra of a 10101×01010 molecular ladder mixture upon dissociation with 1.5 eq of scandium triflate (green) and subsequent binding upon extracting the scandium triflate and annealing at 70°C (purple).

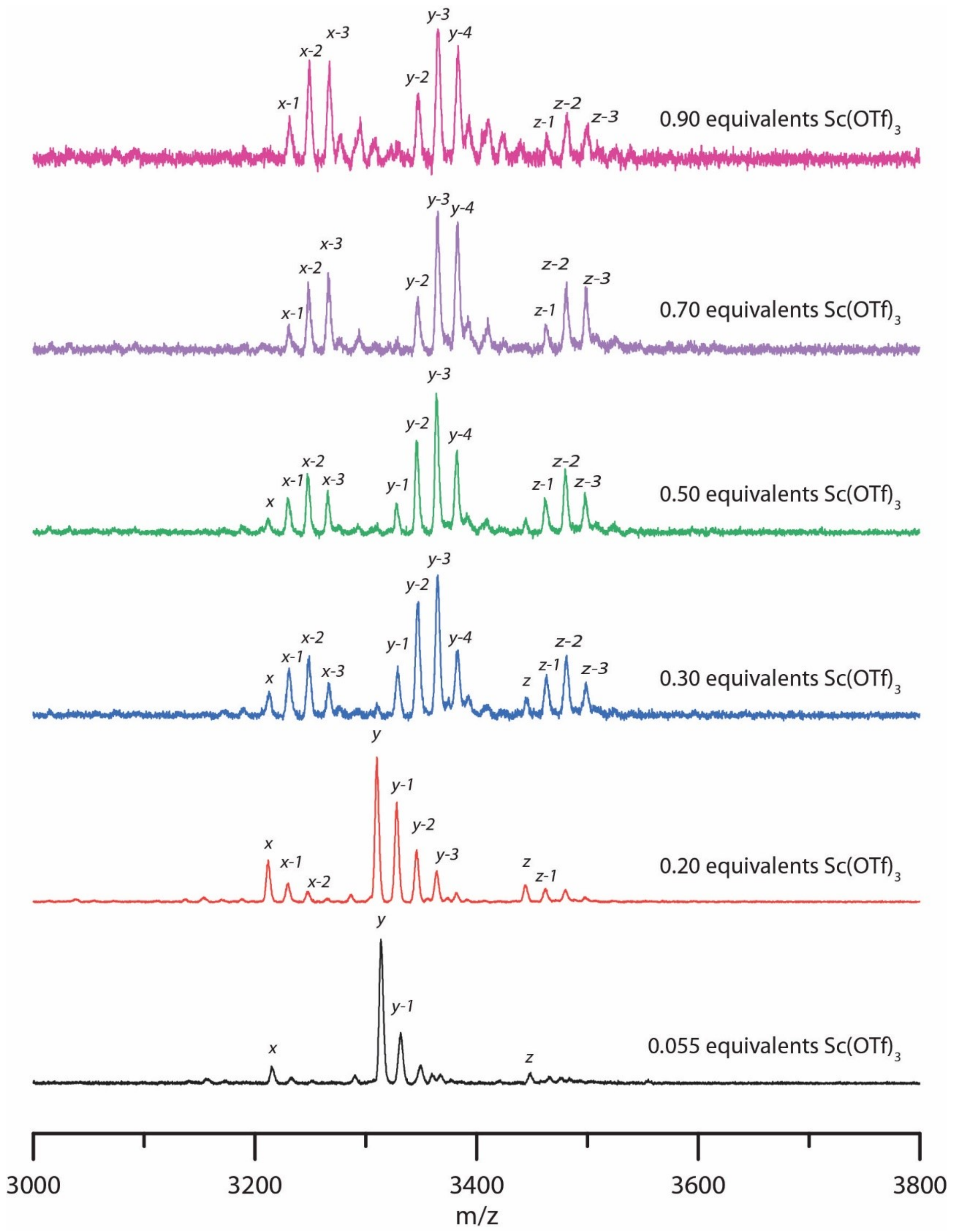


**Supplementary Figure 7** MALDI mass spectra of 10101×01010 molecular ladders treated with additional $Sc(OTf)_3$ post-annealing and allowed to equilibrate. Expected exact masses: $[M_{01010\times01010}+Na]^+$ ($x$) = 3208.63; $[M_{10101\times01010}+Na]^+$ ($y$) = 3306.71; $[M_{10101\times10101}+Na]^+$ ($z$) = 3440.81. Peaks at multiples of +18 m/z values are attributable to ladders species with fewer rungs (e.g., $y$ = in-registry, 5-rung 10101×01010 molecular ladder, $y$-1 = out-of-registry, 4-rung 10101×01010 molecular ladder, etc.)

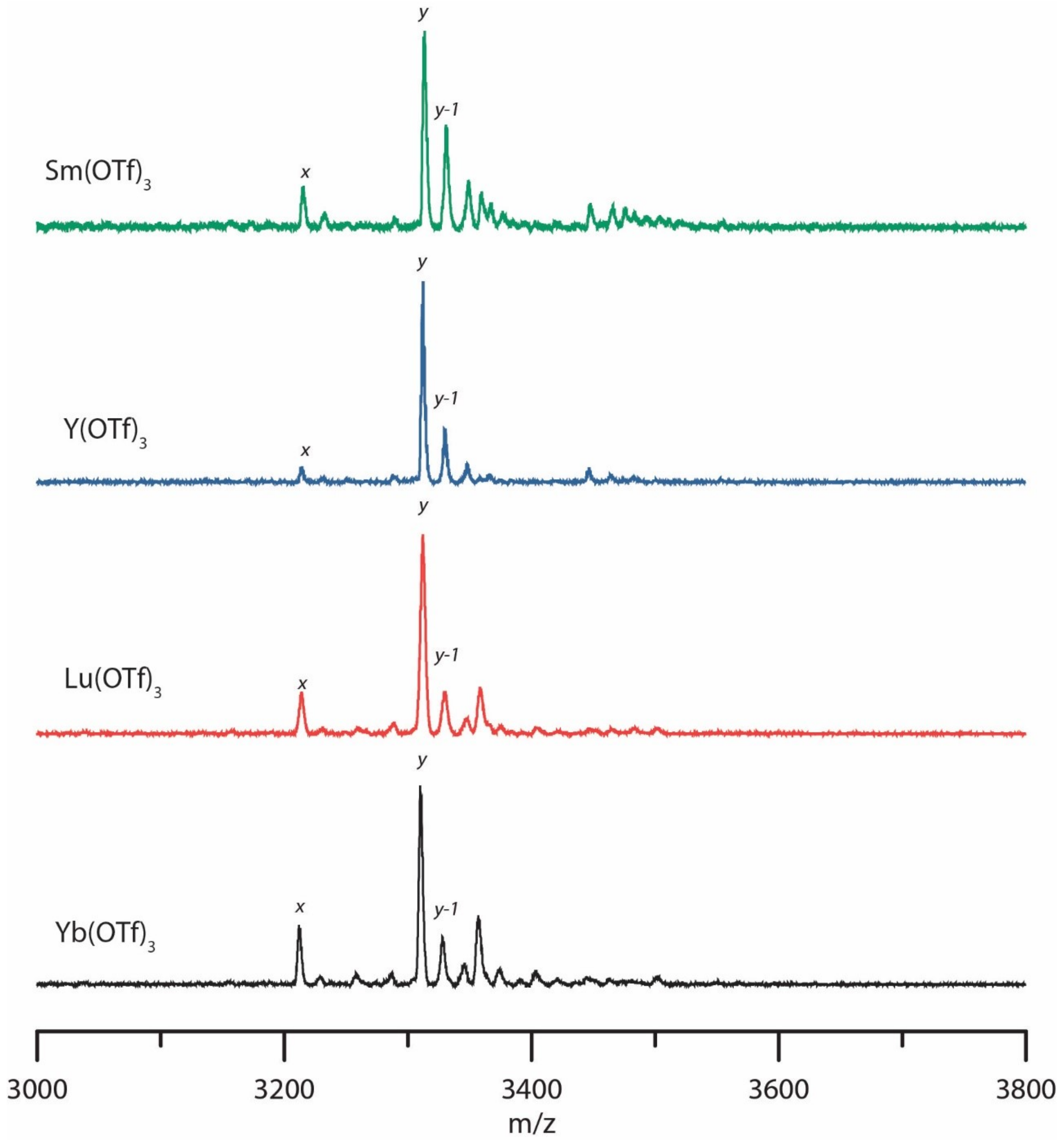


**Supplementary Figure 8** MALDI mass spectra of 10101×01010 molecular ladders *via* the dissociation/extraction/ annealing process challenged against a library of rare-earth metal triflates. Expected exact masses: $[M_{01010\times01010}+Na]^+$ ($x$) = 3208.63; $[M_{10101\times01010}+Na]^+$ ($y$) = 3306.71. Peaks at multiples of +18 m/z values are attributable to ladders species with fewer rungs (e.g., $y$ = in-registry, 5-rung 10101×01010 molecular ladder, $y$-1 = out-of-registry, 4-rung 10101×01010 molecular ladder, etc.)

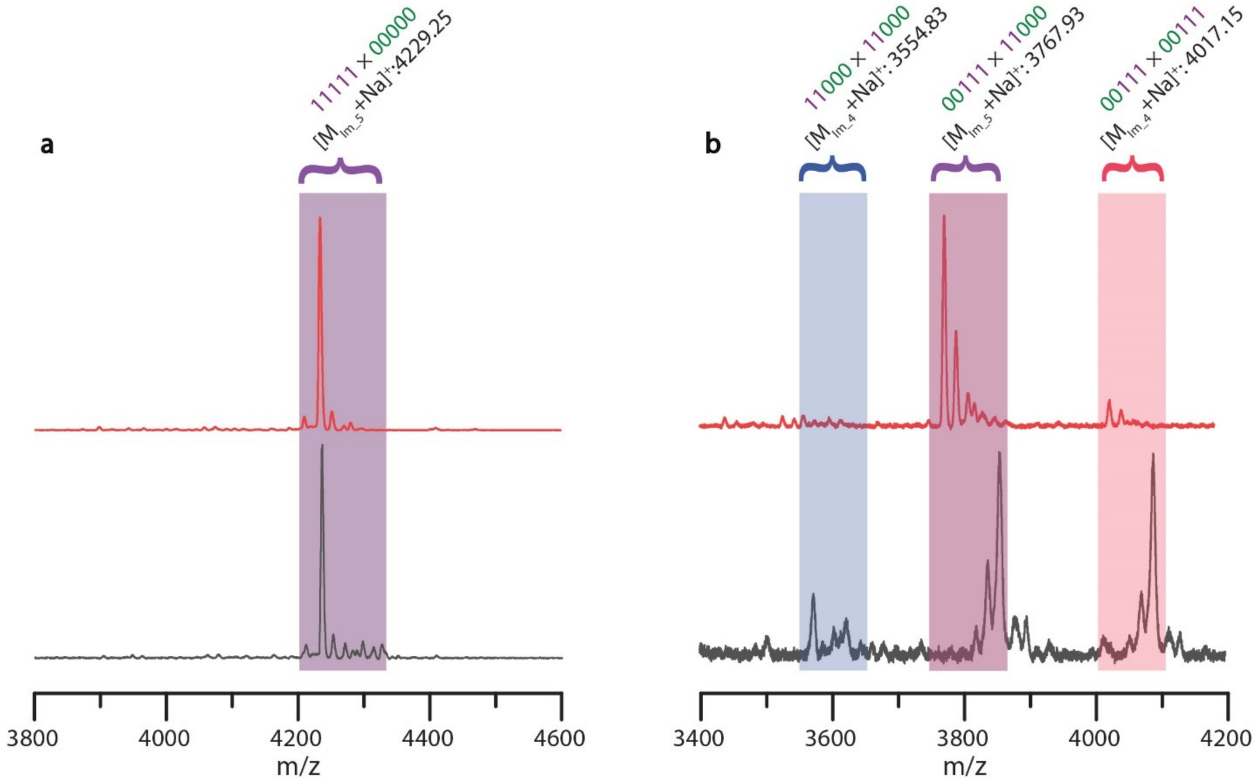


**Supplementary Figure 9** MALDI mass spectra of molecular ladder reaction mixtures utilizing either the previously-determined, single-pot, single-step approach (bottom, black), or *via* the dissociation/extraction/ annealing process (top, red) of **a**, 11111×00000, and **b**, 00111×11000.

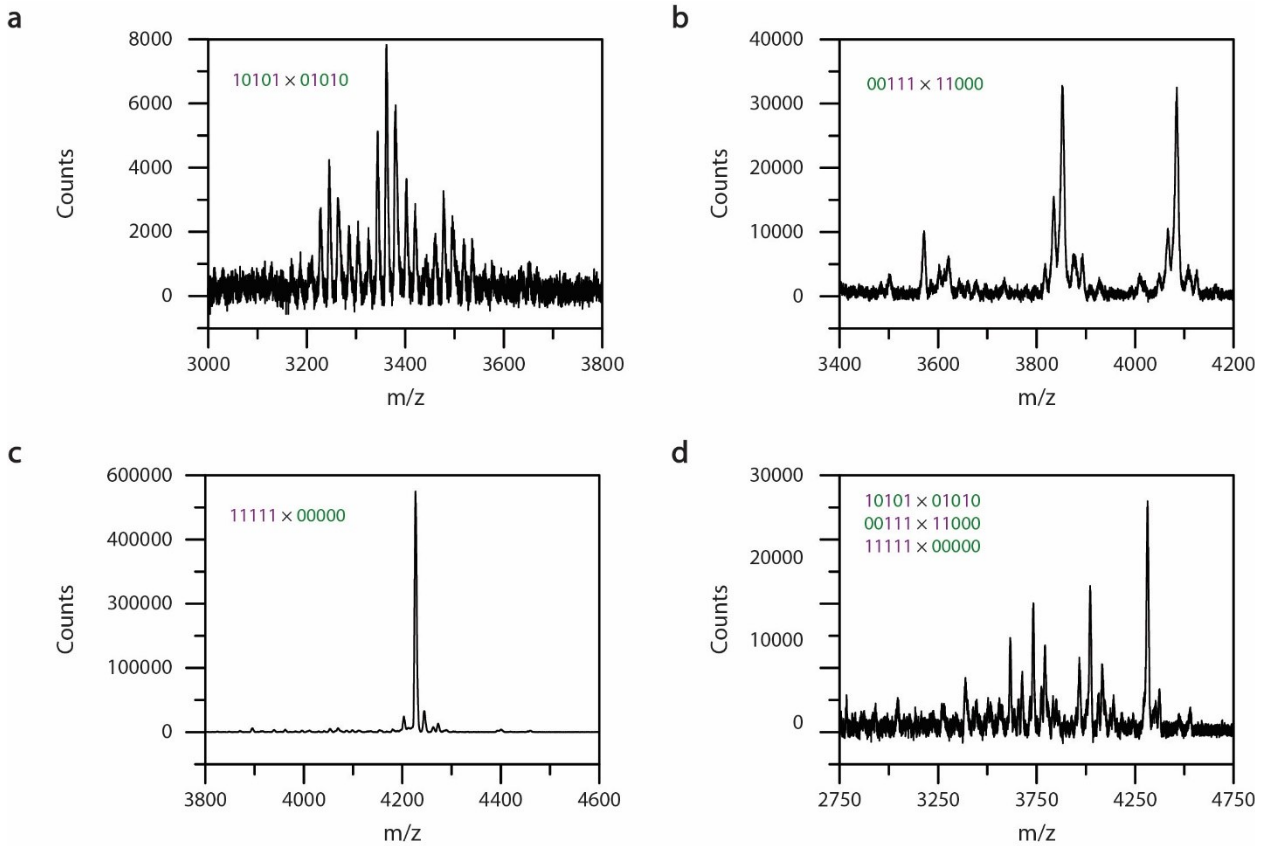


**Supplementary Figure 10** MALDI mass spectra, with counts, of molecular ladder reaction mixtures utilizing the previously-determined, single-pot, single-step approach of **a**, 10101×01010, **b**, 00111×11000, **c**, 11111×00000, and **d**, all six single strands simultaneously.

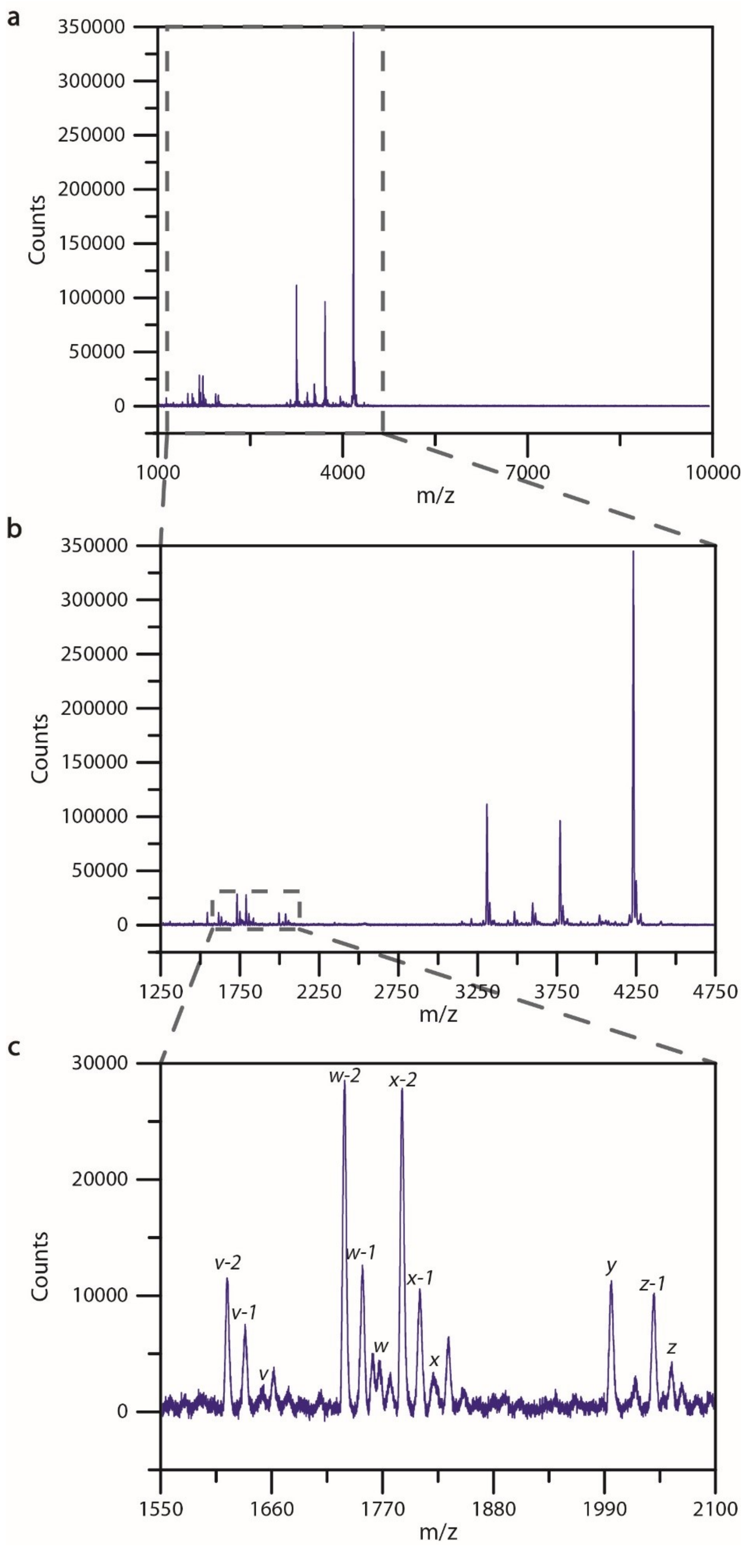


**Supplementary Figure 11** MALDI mass spectrum of a single-pot solution of six oligomers, including 10101, 01010, 00111, 11000, 11111, and 00000, assembled *via* the

dissociation/extraction/annealing process. **a**, m/z 1000-10000 (full range), **b**, m/z 1250-4750 (expected range for dimeric and single-stranded species), and **c**, m/z 1550-2100 (expected range for single-stranded species). Expected exact masses: $[M_{01010}+Na]^+$ (*v*) = 1651.83; $[M_{10101}+Na]^+$ (*w*) = 1767.92; $[M_{11000}+Na]^+$ (*x*) = 1824.93; $[M_{00000}+Na]^+$ (*y*) = 1995.97; $[M_{00111}+Na]^+$ (*z*) = 2056.09. Peaks at multiples of -18 m/z values are attributable to single-stranded species incorporating intramolecular imine bonds (e.g., *v* = 01010, *v*-1 = 01010 with one intramolecular imine bond, *v*-2 = 01010 with two intramolecular imine bonds, etc.).

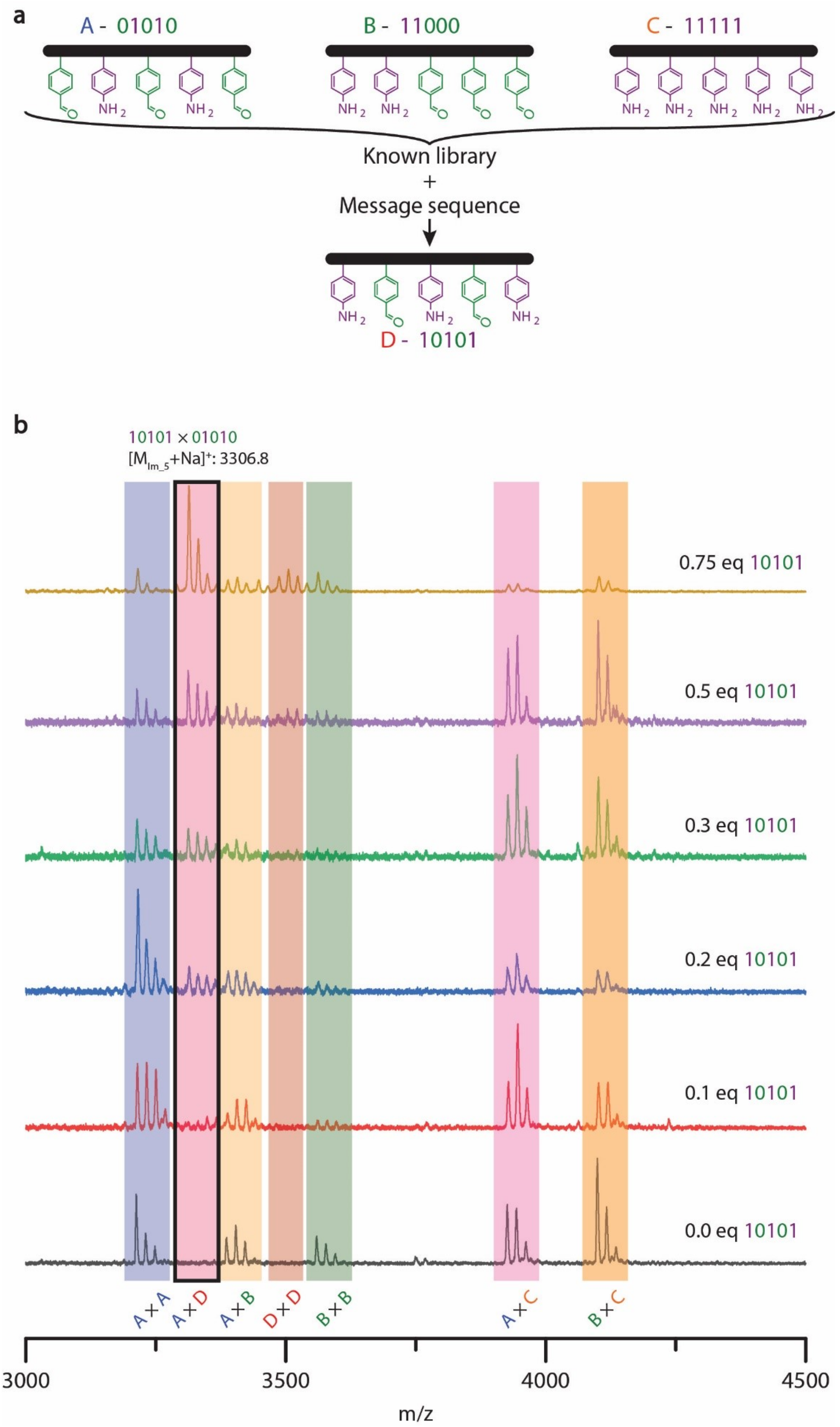


**Supplementary Figure 12** Dynamic covalent information storage and retrieval with varying equivalents of the message strand. **a**, Schematic of a known library composed of three unique,

mass-labeled oligomeric sequences A, B, and C, and individual message sequence used to challenge the library, D. **b**, MALDI mass spectrum of the known sequence library treated to increasing equivalents of the message strand.

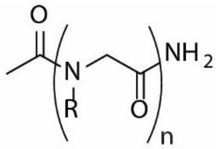

poly-N-substituted glycine

| R = side chain | Designator |
|---|---|
| 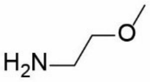 | Nme = 2-methoxyethylamine |
| 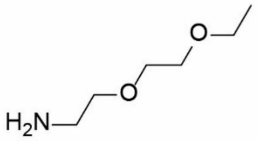 | Neee = 2-(2-ethoxyethoxy)ethylamine |
| 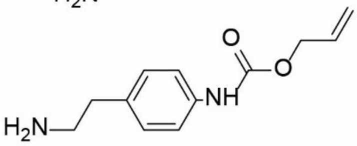 | Npam = 4-(2-aminoethyl)-N-(allylcarbonyloxy)phenylamine |
| 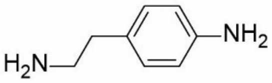 | Nam = 4-(2-aminoethyl)aniline |
| 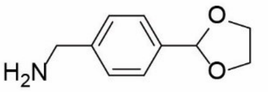 | Npal = 4-(1,3-dioxacyclopent-2-yl)benzylamine |
| 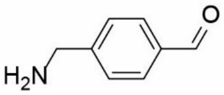 | Nal = 4-(aminomethyl)benzaldehyde |
| 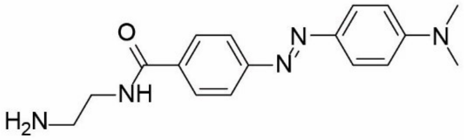 | Ndab= DABCYL-EN |

**Supplementary Table 1** Primary amine monomers used for peptoid synthesis.

| **Known Library** | | **11111 x 00000** | |
|---|---|---|---|
| Sequence Combination | Normalized Intensity | Sequence Combination | Normalized Intensity |
| 01010 x 01010 | 0.65 | 01010 x 01010 | 0.14 |
| 01010 x 11000 | 0.48 | 01010 x 11000 | 0.11 |
| 01010 x 11111 | 0.75 | 01010 x 00000 | - |
| 11000 x 11000 | 0.33 | 01010 x 11111 | - |
| 11000 x 11111 | 1.00 | 11000 x 11000 | 0.08 |
| | | 11000 x 00000 | - |
| | | 11000 x 11111 | - |
| | | 11111 x 00000 | 1.00 |
| **00111 x 11000** | | **10101 x 01010** | |
| Sequence Combination | Normalized Intensity | Sequence Combination | Normalized Intensity |
| 01010 x 01010 | 0.51 | 01010 x 01010 | 0.23 |
| 01010 x 11000 | 0.21 | 01010 x 10101 | 1.00 |
| 01010 x 00111 | 0.45 | 01010 x 11000 | 0.23 |
| 01010 x 11111 | 0.30 | 01010 x 11111 | 0.12 |
| 11000 x 11000 | 0.17 | 10101 x 11000 | 0.36 |
| 11000 x 00111 | 1.00 | 10101 x 10101 | 0.07 |
| 00111 x 00111 | - | 11000 x 11000 | 0.27 |
| 11000 x 11111 | 0.42 | 11000 x 11111 | 0.20 |

**Supplementary Table 2** Hybridization specificity of message strands with their library complements assessed by normalized MALDI mass spectrum intensities.
